# Supplementary material for: Differential impact from individual versus collective misinformation tagging on the diversity of Twitter (X) information engagement and mobility
Source: Nat Commun. 2025 Jan 24;16:973. doi: 10.1038/s41467-025-55868-0 (PMC11760358; doi:10.1038/s41467-025-55868-0)
Supplement: Supplementary file 1 — Supplementary Information [file 41467_2025_55868_MOESM1_ESM.docx]

## **Supplementary Method 1: Additional Analyses regarding the Effects of Collective Tagging on Content Diversity**

Despite the steepness of the slope following collective tagging, our analysis indicates that content diversity does not drop below the pre-tagged period. Specifically, we estimate the significance of pairwise differences between weeks (i.e., comparing Week 5 to Week -5, Week 5 to Week -4, and so on, up to Week 5 versus Week 0). As shown in Supplementary Table 23, although content diversity approaches the level of content diversity observed in the pre-tagged period, it does not significantly decrease below that. For instance, when comparing the content diversity in Week 5 with that in Week -5 (see Fig. 2A), the difference in content diversity between these two points is not significant (*β*=-.001, 95% CI=[-.033, .030], *t*(418,115)=-.09, *p=*.929, *N*=424,969). Similarly, the comparison of content diversity between Week 5 and Week -1 (right before tagging, as depicted in Fig. 2A) shows no significant difference (*β*=-.012, 95% CI=[-.040, .016], *t*(418,115)=-.84, *p=*.398, *N*=424,969).

Nevertheless, some might be concerned that content diversity might eventually drop below the pre-tagged period after Week 5. However, after Week 5, we find that the slope becomes almost flat. Specifically, we find that the slope of change in content diversity between Week 5 and Week 8 becomes close to zero (*β*=.002, 95% CI*=*[-.016, .020]*, t*(11,623)=.25, *p=*.799, *N*=12,056). This means that the original posters maintain their position after reaching the initial level of content diversity at Week 5.

##

## **Supplementary Method 2: Political and Content Proximity to Misinformation Taggers**

We conduct additional analyses to examine how misinformation posters’ political and content proximity to taggers changes after misinformation tagging occurs. Since we could identify only the Twitter accounts of individual misinformation taggers and not those of collective misinformation taggers, our analysis uses data from individual misinformation tagging.

First, we measure political stances of taggers in individual tagging by averaging the MediaBias/FactCheck scores (see Method: Political Diversity) of sources referenced in their historical tweets. This span includes two months before and two months after the posting of tagged tweets. Taggers who predominantly cite left-leaning media are considered “left,” while those who cite right-leaning media are considered “right.” Specifically, taggers with negative average political stance scores are categorized as left, while those with positive scores are categorized as right.

Second, we measure the political proximity between each poster’s tweet and the misinformation tagger. Specifically, we assign a binary value to denote the political proximity: 1 (high proximity) is assigned if the poster cites a source with the same political stance as the tagger; 0 (low proximity) is assigned if not. Similarly, we assess the tweet’s content proximity to the tagger. For this, we calculate the cosine similarity between the embedding of each poster’s tweet and the tagger’s embedding to indicate the content proximity between a specific tweet and the tagger.

As shown in Supplementary Fig. 8 and Supplementary Table 24, we estimate interrupted time series (ITS) models for political and content proximity to taggers. Before individual misinformation tagging, posters increase the political proximity (*β*=.146, 95% CI=[.028, .264], *t*(312333)=2.42, *p=*.015) and content proximity (*β*=.003, 95% CI=[.001, .006], *t*(312333)=2.43, *p=*.015) to taggers. Immediately after tagging, posters significantly decrease the political proximity (*β*=-.536, 95% CI=[-.996, -.077], *t*(312333)=-2.29, *p=*.022), but they do not significantly change content diversity (*β*=.007, 95% CI=[-.004, .018], *t*(312333)=1.27, *p=*.206). After tagging, posters collapse in both political proximity (*β*=-.203, 95% CI=[-.366, -.039], *t*(312333)=-2.43, *p=*.015) and content proximity (*β*=-.005, 95% CI=[-.009, -.001], *t*(312333)=-2.57, *p=*.010) to taggers.

##

## **Supplementary Method 3: Controlling Time-variant Confounders in Interrupted Time Series (ITS) Analyses**

Interrupted Time Series (ITS) analyses assume that no other events capable of affecting the outcome variables (e.g., viral news, platform algorithm changes, or significant external events) coincide with the intervention. To address potential confounding events, we perform additional analyses.

First, we control the occurrence of major events during the study period via sensitivity analyses. As shown in Supplementary Table 25, we identify six major events during our study period; (1) SARS-CoV-2 Omicron variant (November 24, 2021), (2) Russia’s invasion of Ukraine (February 24, 2022), (3) Acquisition of Twitter by Elon Musk (Initiated) (April 14, 2022), (4) Acquisition of Twitter by Elon Musk (Completed) (October 27, 2022), (5) 2022 United States elections (November 8, 2022), and (6) 2023 Turkey-Syria earthquakes (February 6, 2023). For each event, we create a binary variable indicating whether each event already occurred (1) or not (0) when the participants posted tweets before and after tagging. Then, we regress political and content diversity against the occurrence of these events to check whether these events influence the outcomes.

Based on regression analyses shown in Supplementary Table 25, we find that the occurrence of these events indeed shape political or content diversity. For example, the Russian invasion of Ukraine on February 24, 2022 was followed by an increase in political diversity (*β*=1.528, 95% CI=[1.056, 1.999], *t=*6.35, *p*<.001) and content diversity (*β*=-.065, 95% CI=[-.077, -.052], *t=*-10.15, *p*<.001). Similarly, upon initiation of Twitter’s acquisition by Elon Musk, both political diversity (*β*=8.856, 95% CI=[1.797, 15.916], *t=*2.46, *p=*.014) and content diversity (*β*=.410, 95% CI=[.223, .597], *t=*4.31, *p*<.001) increased. These results suggest that surprising events could motivate users to explore previously unexplored, diverse information.

As events in Supplementary Table 25 could potentially confound the effects of misinformation tagging on political and content diversity, we control for the occurrence of these events in our Interrupted Time Series (ITS) analyses. Even after controlling these events, however, the effects of misinformation tagging did not change meaningfully. After individual tagging, both political diversity (*β*=-1.106, 95% CI=[-1.544, -.667], *t*(418109)=-4.94, *p*<.001) and content diversity (*β*=-.027, 95% CI=[-.039, -.016], *t*(418109)=-4.64, *p*<.001) significantly decrease. After collective tagging, however, content diversity significantly increases (*β*=.040, 95% CI=[.011, .069], *t*(418109)=2.70, *p=*.007) (refer to Supplementary Table 26). This supports our main findings that individual misinformation tagging reinforces echo chambers, but collective tagging does not.

Second, we implement comparative interrupted time series (CITS) analyses to provide a more robust baseline for comparison. For each user who posted misinformation, we identify other users who were not yet exposed to misinformation tagging. We then construct a control time series using the average political and content diversity of tweets posted on the same day by these other users.

For example, if a particular user posted a “treated” tweet and was tagged on April 1, the treatment time series in the interrupted time series (ITS) spans five weeks before and after April 1. To create a control time series, we select other users who posted misinformation on the same topic. Then, we calculate the average political and content diversity of their tweets over the same timeframe (i.e., five weeks before and after April 1), ensuring that these users had not posted misinformation and had not been tagged when they posted these tweets. In other words, control time series captures how users would behave without being tagged by individual or collective tags.

Let *Y* represent the outcome variable (political or content diversity of a tweet), *W* the weeks relative to the “treated” tweet (negative for pre-treatment, positive for post-treatment), and *T* an indicator for treatment status (0 for pre-treatment, 1 for post-treatment). *G* distinguishes control (0) and treatment (1) time series and *N* denotes the number of tweets per day (control variable). *α* is the user-fixed effect, and *ϵ* is the error term. The CITS model is specified as follows:

*Y = β_0_  + β_1_W + β_2_T + β_3_WT + β_4_WG + β_5_TG + β_6_WTG + β_7_N + α + ϵ* (1)

Here, *β_4_*, *β_5_*, and *β_6_* capture differences between the control and treatment time series. *β_4_* reflects the difference in slope before tagging, *β_5_* captures the difference in immediate change after tagging, and *β_6_* represents the difference in slope change. For instance, if the “immediate change” in the outcome after tagging is caused by tagging rather than by other concurrent events, we would expect a non-zero, statistically significant coefficient for *β_5_*. We fit the CITS model separately for individual and collective tagging data.

Using the CITS model in Supplementary Table 27, we find that our main findings hold. After receiving individual misinformation tags, users decrease political diversity (*β*=-.905, 95% CI=[-1.344, -.465], *t*(719877)=-4.030, *p*<.001) and content diversity (*β*=-.018, 95% CI=[-.030, -.006], *t*(719877)=-3.01, *p=*.003), which are significant even when compared to control time series. After receiving collective misinformation tagging, users increase content diversity (*β*=.100, 95% CI=[.064, .136], *t*(96120)=5.39, *p*<.001) compared to the control time series.

## **Supplementary Method 4: Autocorrelated Posting Behaviors**

We note that autocorrelated posting behaviors could lead to the underestimation of standard errors in our Interrupted Time Series (ITS) analyses. In other words, political and content diversity of the tweets could be correlated with behaviors in preceding tweets, which could introduce autocorrelation and inflate the statistical significance of the intervention.

Therefore, we conduct the Durbin-Watson test for first-order autocorrelation to examine the presence of autocorrelation in the residuals, which support the presence of autocorrelation for both political diversity (*DW*=1.734, *p*<.001) and content diversity (*DW*=1.615, *p*<.001). Given that autocorrelated posting behaviors are found, we subsequently include autoregressive terms (or lagged dependent variables) in the model to adjust for autocorrelation. Specifically, we add a first-order autoregressive term (i.e., political or content diversity from the immediately prior tweet posted by the user) to the model.

After including autoregressive terms, the results remain consistent. After individual tagging, both political diversity (*β*=-.900, 95% CI=[-1.336, -.464], *t*(411919)=-4.050, *p*<.001) and content diversity (*β*=-.025, 95% CI=[-.036, -.013], *t*(411919)=-4.24, *p*<.001) significantly decrease. After collective tagging, content diversity significantly increases (*β*=.029, 95% CI=[.000, .057], *t*(411919)=1.98, *p=*.047) (refer to Supplementary Table 28).

## **Supplementary Method 5: Propensity Score Weighting**

To control for the distribution of topics, we implement propensity score weighting (PSW) methods^1^. PSW balances the distribution of topics between individual and collective tagging by oversampling tweets corrected by collective tagging if they are topically more likely to be addressed by individual tagging (e.g., if a specific topic is predominantly corrected by individual tagging, the model increases the weight of collective tagging for that topic). Statistically, this technique removes the selection bias based on topics (i.e., different probability of receiving individual versus collective tagging across different topics). This approach helps to control not only the differences between political and non-political misinformation but also the more nuanced topical variations between tweets corrected by each type of tagging.

Specifically, let *C* is an indicator of the type of misinformation tagging where 0 represents individual misinformation tagging and 1 represents collective misinformation tagging. *X_i_* is an indicator of whether a particular tweet belongs to a topic *i*. For instance, the value of *X_i_* is 1 if a particular tag corrects Topic *i* and 0 otherwise. We fit logistic regression that predicts the probability of receiving collective tags, instead of individual tags, on *X_1_*, *X_2_*, ..., *X_9_*.

*log(C / (1-C)) = β_0_ + β_1_X_1_ + β_2_X_2_ + β_3_X_3_ + β_4_X_4_ + β_5_X_5_ + β_6_X_6_ + β_7_X_7_ + β_8_X_8_ + β_9_X_9_ + ε (1)*

Let *P* denote the probability of receiving collective tags based on this model. Then, we estimate the inverse probability weight *IPW* as follows:

*IPW = 1/P if C = 1*

*IPW = 1/(1-P) if C = 0 (2)*

Finally, we apply inverse probability weights (IPW) to adjust our interrupted time series (ITS) models. By applying these weights, we control for any differences in the outcomes between individual and collective tags introduced by topical differences of the corrected misinformation.

In Supplementary Table 29, we show that PSW balances the distribution of topics between tweets fact-checked by individual and collective tags. Furthermore, in Supplementary Fig. 9 Panel A, we extract 768-dimensional semantic embeddings from the tagged tweets using Twitter4SSE, which capture nuanced semantic differences not captured by topics. In Supplementary Fig. 9, Panel B, we measure the average standardized difference between “tweets tagged by individual tags” and “tweets tagged by collective tags” before and after PSW. This standardized difference is calculated for each dimension of the semantic embeddings, and the average across all dimensions is reported. The results suggest that the average standardized difference decreases from .227 to .177 (Difference=-.050, 95% CI=[.041, .059], *t*(767)=11.27, *p*<.001), reducing semantic differences between individual and collective tagging.

## **Supplementary Method 6: Political Stances of Taggers and Voters**

We analyze the distribution of political stance among taggers and voters in each condition. Taggers refer to users who write individual tags (i.e., fact-checking replies), and voters refer to users who vote on the exposure of collective tags. Note that we have voters only in collective tagging because individual tags are exposed without votes. Here, we have four conditions: individual tags correcting left-leaning users, individual tags correcting right-leaning users, collective tags correcting left-leaning users, collective tags correcting right-leaning users. For each condition, the distribution of taggers’ and voters’ political stances are shown in Supplementary Table 9.

In every condition, we find that left-leaning taggers and voters are more prevalent. Compared to individual tagging, community notes are more balanced as they consider more right-leaning perspectives. Still, the majority of votes come from left-leaning voters. Based on this imbalance, we suspect that right-leaning voters are more likely to be “filtered” due to their low-quality contributions in the past (see Supplementary Method 7: Potential Backlash Among Voters in Community Notes). Therefore, right-leaning posters are likely to be corrected by someone of different political stances (i.e., left-leaning taggers and voters) in both individual and collective tagging.

We measure taggers’ and voters’ proxy political stances as follows. First, political stances of taggers in individual tagging is measured by averaging the MediaBias/FactCheck scores (see Method: Political Diversity) of sources referenced in their historical tweets. This span includes two months before and two months after the posting of tagged tweets. Taggers who predominantly cite left-leaning media are considered “left,” while those who cite right-leaning media are considered “right.” Specifically, taggers with negative average political stance scores are categorized as left, while those with positive scores are categorized as right.

Second, political stances of voters in collective tagging are measured by their activities in Community Notes. We were not able to access these anonymous voters’ historical tweets to measure their political stances, but we were able to fully access their activities in Community Notes. Allen and colleagues (2024) imply that voters’ activities in Community Notes can be a proxy of their political stances, documenting a strong correlation between voters’ political stances and their activities^2^. Therefore, we assume that voters are left-leaning if their prior activities in Community Notes show they “tag” or “vote” on right-leaning politicians’ tweets as misinformation more frequently than those of left-leaning politicians. Conversely, we assume that voters are right-leaning if their prior records indicate they tag or vote on left-leaning politicians’ tweets as misinformation more often than those of right-leaning politicians. We exclude voters who have never tagged or voted on politicians’ tweets when estimating voters’ political stances or filtering tags in which left-leaning voters correct right-leaning posters, as shown in Supplementary Tables 9 and 10. We use the list of left-leaning and right-leaning politicians aggregated by Barberá (2015)^3^.

## **Supplementary Method 7: Potential Backlash Among Voters in Community Notes**

Some may concern that voters from one political party can engage in backlash in Community Notes, obstructing the correction of misinformation widespread within that party (e.g., misinformation about vaccination or the 2020 election) by collectively “disagreeing” with the correction. However, we find that backlash may be an exception, rather than the norm. Community notes evaluate cross-perspective agreements^4^, but they do so only after filtering out voters likely to backlash and oppose valid fact-checks. Specifically, the algorithm assesses whether users’ votes are not aligned with the crowd’s final decisions by more than 33% and removes their votes^4^. In other words, if a voter casted 10 votes in community notes, and more than 3 votes were found to be different from the crowd’s decisions, their votes are excluded by the algorithm. Due to the high threshold to be eligible voters, users who misuse their votes to obstruct valid corrections are likely to be disregarded.

Indeed, almost all eligible voters are found not to *veto* vaccine- and election-related collective tags. We first have identified 3,558 vaccine-related tags containing the word “vaccine,” and 2,597 election-related tags containing the word “election” in Community Notes. Among 4,535 voters who passed algorithmic filtering in Community Notes, 96.3% have never opposed vaccine-related tags, and 98.54% have not opposed election-related tags approved by the crowd. This suggests that community notes require cross-perspective agreements within a moderate (not extreme) political population who rarely disagree with valid fact-checks against blatant misinformation, such as COVID-19 and election misinformation.

##

## **Supplementary Method 8: Inter-Rater Reliability for Political Stances Derived from MediaBias/FactCheck**

We refer to the political stance scores from the MediaBias/FactCheck (MBFC) database (<https://mediabiasfactcheck.com/>) to assess each tweet’s political stance. Specifically, for tweets with political scores ranging from -1 (extreme left) to 0, we label them as “left”, and for tweets with political scores ranging from 0 to 1 (extreme right), we label them as “right”.

To address the concerns regarding potential biases from the MediaBias/FactCheck scores, we estimate the inter-rater reliability of MBFC scores with an alternative database from [Allsides.com](http://allsides.com), which labels 445 websites as “left,” “leaning left,” “leaning right,” or “right” (we exclude websites labeled as “center” or “mixed”)^5^. We map these labels to “left” (including “left” and “leaning left”) and “right” (including “right” and “leaning right”). Then we calculate the inter-rater reliability scores between MBFC and [Allsides.com](http://allsides.com) on 257 websites (covered in both databases) using Cohen’s Kappa and get a reliability score of .9161, which indicates the substantial agreements between the two databases.

## **Supplementary Method 9: Limiting the Sample to Corrective Individual Tags**

We find that individual tags (i.e., PolitiFact links) are mostly used to correct the original posters’ arguments. To strictly identify PolitiFact links that correct the original posters, we submitted original posts, replies containing PolitiFact links, and the cited PolitiFact fact-checking articles to ChatGPT (gpt-4o-2024-05-13). We prompted the model to annotate whether the PolitiFact link was used to correct the original poster, rather than support them. Specifically, we utilized the following prompt template.

*Here is a tweet and its reply. The reply includes a link to a fact-checking article from PolitiFact. Does the reply use the article to correct the original tweet (rather than support it)?*

*[OP's tweet] {Content of the tagged tweet}*

*[Reply] {Content of the fact-checking message}*

*[Fact-checking article cited in the reply] {Claim date, speaker, claim, and rating information from the PolitiFact article}*

*Determine whether the reply or the fact-checking article corrects the original tweet. Simply respond with 1 if it does, and 0 if it does not or if it is not clear.*

Consequently, we identified 5,592 politifact links out of 6,760 links (82.72%) that we can confidently say that they are corrective. To assess the accuracy of this annotation method, the researcher performed the same task on a randomly selected sample of 50 PolitiFact links. The results revealed high accuracy between the human annotator and ChatGPT, with an F1 score of 85.2%, precision of 92.0%, and recall of 79.3%. We find that the precision is higher than the recall, which means that ChatGPT uses stricter and more conservative criteria to determine whether the PolitiFact link corrects the original poster.

For instance, ChatGPT responds that the following reply and PolitiFact link corrects the original poster (i.e., 1).

*Here is a tweet and its reply. The reply includes a link to a fact-checking article from PolitiFact. Does the reply use the article to correct the original tweet (rather than support it)?*

*[OP's tweet] We’ve seen almost double the number of children pass away from the vaccine compared to those lost to COVID. [Link]*

*[Reply] @user1 @user2 That’s not true. https://t.co/47fIls9Xfc*

*[Fact-checking article cited in the reply]*

*Claim Date: 2021-11-05*

*Speaker: Viral image (Posters on Facebook and other social media; Party: None)*

*Claim: “Children are 50 times more likely to be killed by the Covid vaccines than by the virus itself.”*

*Rating: Pants on Fire*

*Determine whether the reply or the fact-checking article corrects the original tweet. Simply respond with 1 if it does, and 0 if it does not or if it is not clear.*

On the other hand, ChatGPT responds that the following PolitiFact link does not correct the original poster (i.e., 0). Indeed, the PolitiFact article is not utilized to correct the original poster.

*Here is a tweet and its reply. The reply includes a link to a fact-checking article from PolitiFact. Does the reply use the article to correct the original tweet (rather than support it)?*

*[OP’s tweet] Why schools in India are failing children on climate change*

*[Link]*

*[Reply] @user1 In the USA.. CLIMATE... over and over.. [Link]*

*[Fact-checking article cited in the reply]*

*Claim Date: 2017-01-17*

*Speaker: Chad Mayes (Party: Republican)*

*Claim: California has “the highest poverty rate in the nation” when considering the U.S. Census Bureau’s Supplemental Poverty Measure.*

*Rating: True*

*Determine whether the reply or the fact-checking article corrects the original tweet. Simply respond with 1 if it does, and 0 if it does not or if it is not clear.*

Subsequently, we limited the sample to the aforementioned 5,592 links identified by ChatGPT from the individual tagging data, which do not meaningfully alter the results (see Supplementary Tables 14 and 15)

## **Supplementary Tables and Figures**

**Supplementary Table 1. Illustrative Examples of Content Diversity Scores for a Sample User.**

|  | Least Diverse Contents | | Most Diverse Contents | |
| --- | --- | --- | --- | --- |
| Rank | Tweet | Content Diversity | Tweet | Content Diversity |
| 1 | Breaking News: Recently released Australian Government reports indicate a significant increase in excess deaths, up to 5162%, compared to the year 2020, potentially linked to COVID vaccination. @user @user, can you provide more insights? [Link to article removed] | .175936 | A call to release Pelosi's tax returns, citing precedent set by Trump's tax return release. #Transparency [Link to article removed] | .505400 |
| 2 | A government study reveals that 92% of COVID deaths were among those who were 'fully vaccinated.' Surprising information not widely covered in the media. [Link to article removed] | .196608 | Miley Cyrus donates to an LGBTQ organization following the banning of her song 'Rainbowland' in an elementary school. [Link to article removed] | .473959 |
| 3 | Japanese experts express confusion over high 'COVID deaths' despite a high vaccination rate, highlighting the need for more information. [Link to article removed] | .204821 | Zambia receives a 'debt-for-nature' proposal from WWF for $13 billion restructuring, raising questions about the motivations behind such deals. [Link to article removed] | .461800 |
| 4 | A German doctor receives a two-year jail sentence for illegally issuing thousands of mask exemptions, sparking a debate on the fairness of such punishment. [Link to article removed] | .205890 | An appreciation of superstars who use their influence for positive impact on humanity. [Link to TikTok video removed] | .456110 |
| 5 | Reports suggest an increase in excess mortality in Australia, but authorities remain quiet on the matter. #HealthCrisis [Link to article removed] | .209318 | Labor approves $9.5 million for 'facts of the voice' without acknowledging it as funding for a de-facto 'yes' campaign. @user, it's crucial for MPs to engage with constituents on this issue. [Link to article removed] | .454970 |

*Notes: The table illustrates the examples of how content diversity scores are measured for a particular user. The user had primarily demonstrated interests centered around COVID-19 and associated misinformation. However, as the user explored a more diverse range of topical interests—including tax, LGBTQ+, international issues, and labor—the content diversity score increased. Following Twitter’s privacy policy, we provide the rephrased tweets using ChatGPT (GPT-3.5) to ensure that user identities remain confidential (Prompt: Rewrite these tweets in a way that preserves the original meaning while respecting user privacy.).*

**Supplementary Table 2. Pairwise Correlation Coefficients Among Variables**

| **Variables** | **Mean** | **Standard Deviation** | **Pairwise Correlation Coefficients** | | | |
| --- | --- | --- | --- | --- | --- | --- |
|  |  |  | **1. Political Diversity (%)** | **2. Content Diversity** | **3. # of tweets per day** | **4. % of retweets per day** |
| 1. Political Diversity (%) | 16.570 | 37.181 |  |  |  |  |
| 2. Content Diversity | .357 | .109 | .020***  [.018, .022]  *t*=16.89  *p*<.001 |  |  |  |
| 3. # of tweets per day | 18.116 | 30.196 | -.107***  [-.109, -.105]  *t*=−90.87 *p*<.001 | −.052***  [−.054, −.050] *t*=−43.97  *p*<.001 |  |  |
| 4. % of retweets per day | 32.650 | .407 | −.046***  [−.048, −.044]  *t*=-38.88 *p*<.001 | .012***  [.010, .014] *t*=10.13  *p*<.001 | −.200***  [−.202, −.198] *t*=−172.35  *p*<.001 |  |
| 5. Low-Credibility Sources (%) | 27.15 | 44.472 | −.227***  [−.229, −.225]  *t*=−196.81 *p*<.001 | −.030***  [−.032, −.028] *t*=−25.34  *p*<.001 | .172***  [.170, .174] *t*=147.43  *p*<.001 | −.041***  [−.043, −.039] *t*=−34.65  *p*<.001 |
| Observations | 712,948 | | | | | |
| *df* | 712,946 | | | | | |

*Notes: ***p<.001. The statistical significance of pairwise correlation coefficients is tested using two-sided t-tests. Confidence intervals (95%) are provided in brackets, along with the corresponding t-statistics, degrees of freedom, and exact p-values. 95% confidence intervals estimated using Fisher Z-transformation are in brackets. df denotes degrees of freedom. Political diversity has been multiplied by 100 so that the estimates are interpretable as absolute percentage point changes. Statistical significance levels (P values) are derived from two-sided tests.*

**Supplementary Table 3. Control Analyses for the Proportion of Retweets**

| **Outcome** | **Political diversity (%)** | | **Content diversity (z)** | |
| --- | --- | --- | --- | --- |
| **Controls** | **Initial results**  **(N = 424,969)** | **Control the proportion**  **of retweets**  **(N = 424,969)** | **Initial results**  **(N = 424,969)** | **Control the proportion**  **of retweets**  **(N = 424,969)** |
| Difference (Collective - Individual) |  |  |  |  |
| Slope before | .072  [-.219, .363]  *t=*.480, *p=*.628 | .100  [-.191, .391]  *t=*.67, *p=*.501 | -.005  [-.012, .003]  *t=*-1.17, *p=*.243 | -.005  [-.013, .003]  *t=*-1.25, *p=*.213 |
| Immediate change | 1.279*  [.101, 2.457]  *t=*2.13, *p=*.033 | 1.278*  [.101, 2.455]  *t=*2.13, *p=*.033 | .070***  [.039, .102]  *t=*4.44, *p*<.001 | .071***  [.039, .102]  *t=*4.44, *p*<.001 |
| Slope after | -.136  [-.440, .167]  *t=*-.88, *p=*.379 | -.124  [-.427, .180]  *t=*-.80, *p=*.424 | -.008*  [-.016, .000]  *t=*-2.01, *p=*.044 | -.008*  [-.016, .000]  *t=*-2.05, *p=*.041 |
| Slope change | -.208  [-.629, .213]  *t=*-.97, *p=*.332 | -.224  [-.644, .197]  *t=*-1.04, *p=*.297 | -.004  [-.015, .007]  *t=*-.64, *p=*.520 | -.003  [-.015, .008]  *t=*-.61, *p=*.539 |
| *R^2^* | .173 | .173 | .243 | .243 |
| Adjusted *R^2^* | .159 | .160 | .230 | .231 |
| Observations | 424,969 | 424,969 | 424,969 | 424,969 |
| *df* | 418,115 | 418,114 | 418,115 | 418,114 |

*Notes: ***p<.001 **p<.01 *p<.05. We multiply political diversity by 100 to interpret the estimates as absolute percentage point changes. We normalize content diversity to z-scores (the number of standard deviations from the mean). All regressions control for user fixed effects and the number of tweets per day. The statistical significance of regression coefficients is tested using two-sided t-tests. Confidence intervals (95%) are provided in brackets, along with the corresponding t-statistics, degrees of freedom, and exact p-values. More details can be found in Methods: Interrupted Time Series (ITS) Analysis.*

**Supplementary Table 4. Descriptive Statistics of Linguistic Characteristics.**

| **Type of Misinformation Tagging** | **Individual** | **Collective** | **Difference (Collective - Individual)** | Observations |
| --- | --- | --- | --- | --- |
| Toxicity (0~1) | .139 (.183) | .076 (.078) | -.063*** [-.076, -.051]  *t*(7496)=-9.86, *p*<.001  Cohen’s *d*=-.228 | 7,498 |
| Sentiment (-1~1) | -.082 (.439) | -.050 (.382) | .032* [.003, .061]  *t*(7731)=2.14, *p=*.033  Cohen’s *d*=.049 | 7,733 |
| Length (Number of Characters) | 179.31 (115.95) | 288.87 (135.28) | 109.56*** [101.59, 117.53]  *t*(7731)=26.95, *p*<.001  Cohen’s *d*=.613 | 7,733 |
| Reading Ease |  |  |  |  |
| 5th grade | .39% | .51% | *χ2*(7)=155.32***, *p*<.001  Cramer’s *V*=.155 | 6,493 |
| 6th grade | 3.17% | 1.37% |  |  |
| 7th grade | 9.04% | 2.39% |  |  |
| 8-9th grade | 16.01% | 8.19% |  |  |
| 10-12th grade | 17.86% | 11.77% |  |  |
| College | 31.56% | 34.64% |  |  |
| College graduate | 16.03% | 27.82% |  |  |
| Professional | 5.94% | 13.31% |  |  |
| Delay (# Days) | 3.037 (42.330) | 6.322 (60.315) | 3.285* [.261, 6.309]  *t*(7731)=2.13, *p=*.033  Cohen’s *d*=.048 | 7,733 |

*Notes: ***p<.001 **p<.01 *p<.05. The values present the mean value or proportion with the standard deviation in parentheses. 95% confidence intervals are in brackets. Significance of difference is estimated by independent two-sample t-test (continuous variable) or chi-square test (categorical variable). The effect size is estimated using Cohen’s d or Cramer’s V. Statistical significance levels (P values) are derived from two-sided tests.*

**Supplementary Table 5. Comparing the Effects of Individual and Collective Tags with Similar Linguistic Characteristics**

| **Outcome** | **Political diversity (%)** | | | | | |
| --- | --- | --- | --- | --- | --- | --- |
| **Linguistic Characteristics** | **Initial results**  **(N=424,969)** | **Non-toxic tags**  **(N=381,552)** | **Neutral tags**  **(N=160,451)** | **Short tags**  **(N=405,829)** | **Easy tags**  **(N=167,766)** | **Quick tags**  **(N=364,024)** |
| Difference (Collective - Individual) |  |  |  |  |  |  |
| Slope before | .072  [-.219, .363]  *t=*.480, *p=*.628 | -.128  [-.565, .308] *t=*-.58, *p=*.564 | .148  [-.152, .447] *t=*.97, *p=*.334 | .163  [-.158, .485] *t=*.99, *p=*.320 | -.057  [-.390, .275] *t=*-.34, *p=*.736 | -.028  [-.412, .356] *t=*-.14, *p=*.886 |
| Immediate change | 1.279*  [.101, 2.457]  *t=*2.13, *p=*.033 | 1.809*  [.029, 3.590] *t=*1.99, *p=*.046 | 1.245*  [.035, 2.455] *t=*2.02, *p=*.044 | 1.071  [-.231, 2.373] *t=*1.61, *p=*.107 | 1.320  [-.037, 2.677] *t=*1.91, *p=*.057 | 1.575*  [.045, 3.104] *t=*2.02, *p=*.044 |
| Slope after | -.136  [-.440, .167]  *t=*-.88, *p=*.379 | .052  [-.404, .508] *t=*.22, *p=*.822 | -.129  [-.440, .182] *t=*-.81, *p=*.417 | -.022  [-.357, .312] *t=*-.13, *p=*.896 | -.022  [-.374, .330] *t=*-.12, *p=*.902 | -.152  [-.545, .240] *t=*-.76, *p=*.447 |
| Slope change | -.208  [-.629, .213]  *t=*-.97, *p=*.332 | .181  [-.450, .811] *t=*.56, *p=*.574 | -.276  [-.708, .156] *t=*-1.25, *p=*.210 | -.185  [-.650, .279] *t=*-.78, *p=*.434 | .035  [-.449, .520] *t=*.14, *p=*.886 | -.124  [-.675, .427] *t=*-.44, *p=*.659 |
| *R^2^* | .173 | .215 | .258 | .174 | .171 | .175 |
| Adjusted *R^2^* | .159 | .203 | .246 | .160 | .158 | .160 |
| Observations | 424,969 | 381,552 | 160,451 | 405,829 | 332,647 | 364,024 |
| *df* | 418,115 | 375,531 | 157,878 | 399,236 | 327,148 | 357,828 |
| **Outcome** | **Content diversity (z)** | | | | | |
| **Linguistic Characteristics** | **Initial results**  **(N=424,969)** | **Non-toxic tags**  **(N=381,552)** | **Neutral tags**  **(N=160,451)** | **Short tags**  **(N=405,829)** | **Easy tags**  **(N=167,766)** | **Quick tags**  **(N=364,024)** |
| Individual vs. collective |  |  |  |  |  |  |
| Slope before | -.005  [-.012, .003] *t=*-1.17, *p=*.243 | -.006  [-.014, .002] *t=*-1.41, *p=*.158 | .011*  [.000, .022] *t=*1.98, *p=*.047 | -.003  [-.011, .006] *t=*-.64, *p=*.519 | -.004  [-.013, .005] *t=*-.94, *p=*.346 | -.015**  [-.025, -.005] *t=*-2.85, *p=*.004 |
| Immediate change | .070***  [.039, .102] *t=*4.44, *p*<.001 | .075***  [.043, .107] *t=*4.63, *p*<.001 | .074**  [.028, .119] *t=*3.20, *p=*.001 | .072***  [.038, .107] *t=*4.11, *p*<.001 | .067***  [.031, .103] *t=*3.68, *p*<.001 | .106***  [.065, .147] *t=*5.09, *p*<.001 |
| Slope after | -.008*  [-.016, .000] *t=*-2.01, *p=*.044 | -.008*  [-.016, .000] *t=*-1.97, *p=*.049 | -.022***  [-.034, -.011] *t=*-3.79, *p*<.001 | -.010*  [-.019, -.001] *t=*-2.16, *p=*.031 | -.016**  [-.025, -.007] *t=*-3.43, *p=*.001 | -.020***  [-.031, -.010] *t=*-3.76, *p*<.001 |
| Slope change | -.004  [-.015, .007] *t=*-.64, *p=*.520 | -.003  [-.014, .009] *t=*-.44, *p=*.661 | -.034***  [-.050, -.018] *t=*-4.11, *p*<.001 | -.007  [-.019, .005] *t=*-1.11, *p=*.269 | -.012  [-.025, .001] *t=*-1.84, *p=*.066 | -.005  [-.020, .010] *t=*-.69, *p=*.488 |
| *R^2^* | .243 | .215 | .257 | .241 | .257 | .238 |
| Adjusted *R^2^* | .230 | .203 | .246 | .228 | .245 | .225 |
| Observations | 424,969 | 381,552 | 160,451 | 405,829 | 332,647 | 364,024 |
| *df* | 418,115 | 375,531 | 157,878 | 399,236 | 327,148 | 357,828 |

*Notes: ***p<.001 **p<.01 *p<.05. We multiply political diversity by 100 to interpret the estimates as absolute percentage point changes. We normalize content diversity to z-scores (the number of standard deviations from the mean). All regressions control for user fixed effects and the number of tweets per day. The statistical significance of regression coefficients is tested using two-sided t-tests. Confidence intervals (95%) are provided in brackets, along with the corresponding t-statistics, degrees of freedom, and exact p-values. More details can be found in Methods: Interrupted Time Series (ITS) Analysis.*

**Supplementary Table 6. Diversity of Topics Corrected by Individual and Collective Tagging**

|  | Shannon H | Exp(H) | 1/Simpson D |
| --- | --- | --- | --- |
| Individual Tagging | 2.336 | 10.344 | 6.697 |
| Collective Tagging | 2.724 | 15.247 | 12.579 |

*Notes: Results show that collective tagging corrects more diverse topics across various metrics compared to individual tagging. To examine whether each tag type selectively and repeatedly corrects certain political topics (low diversity) or corrects diverse topics (high diversity), we use Shannon H (Shannon Diversity Index), Exp(H) (Exponent of Shannon Index), and 1/Simpson D (Inverse Simpson Diversity Index). Shannon H is measured by −∑(p_i_log(⁡p_i_)), where p_i_​ is the proportion of tags belonging to a particular topic, with higher values indicating higher diversity. Simpson D is measured by ∑(p_i_^2^​). Higher values of 1/Simpson D indicate higher diversity.*

**Supplementary Table 7. Most Frequent Topics Corrected by Individual and Collective Tagging**

| **Topic ID** | **Top keywords** | **Frequency** | **Total (%)** | **Individual (%)** | **Collective (%)** |
| --- | --- | --- | --- | --- | --- |
| 1 | covid, vaccine, vaccines, vaccinated | 1,753 | 26.32% | 28.42% | 10.26% |
| 2 | fact, just, lies, true | 1,295 | 19.44% | 20.76% | 9.35% |
| 3 | gun, state, state lines, lines | 554 | 8.32% | 8.42% | 7.53% |
| 4 | projection, lie republican, monster, fyi | 491 | 7.37% | 7.91% | 3.25% |
| 5 | tax, biden, inflation, bernie | 487 | 7.31% | 7.67% | 4.55% |
| 6 | ukraine, russia, biden, putin | 218 | 3.27% | 2.84% | 6.62% |
| 7 | votes, election, voters, vote | 216 | 3.24% | 3.6% | 0.52% |
| 8 | hillary, thomas, clinton, just | 199 | 2.99% | 3.06% | 2.47% |
| 9 | turkey, earthquake, climate, al gore | 196 | 2.94% | 1.38% | 14.94% |
| Total | | 5,409 | 81.20% | 84.06% | 59.49% |

*Notes: Top keywords have been identified by counting the most frequent words within tweets corresponding to each topic, following the exclusion of stopwords. Total (%) column indicates the proportion of a particular topic in relation to all corrected misinformation. Individual (%) column indicates the proportion of a particular topic out of the misinformation corrected through individual tagging. Collective (%) column indicates the proportion of a particular topic out of the misinformation corrected through collective tagging.*

**Supplementary Table 8. Comparing the Effects of Individual and Collective Tags Targeting Similar Topics of Tweets**

| **Outcome** | **Political diversity (%)** | | | **Content diversity (z)** | | |
| --- | --- | --- | --- | --- | --- | --- |
| **Controls** | **Initial results**  **(N = 424,969)** | **Limiting topics**  **(N = 301,380)** | **Propensity score weighting**  **(N = 301,380)** | **Initial results**  **(N = 424,969)** | **Limiting topics**  **(N = 301,380)** | **Propensity score weighting**  **(N = 301,380)** |
| Difference (Collective - Individual) |  |  |  |  |  |  |
| Slope before | .072  [-.219, .363]  *t=*.480, *p=*.628 | -.250  [-.658, .157] *t=*-1.20, *p=*.229 | -.409  [-.952, .134] *t=*-1.48, *p=*.140 | -.005  [-.012, .003] *t=*-1.17, *p=*.243 | .002  [-.009, .013] *t=*.36, *p=*.721 | -.007  [-.018, .005] *t=*-1.16, *p=*.244 |
| Immediate change | 1.279*  [.101, 2.457]  *t=*2.13, *p=*.033 | 1.999*  [.343, 3.655] *t=*2.37, *p=*.018 | 2.380*  [.200, 4.560] *t=*2.14, *p=*.032 | .070***  [.039, .102] *t=*4.44, *p*<.001 | .036  [-.008, .079] *t=*1.62, *p=*.105 | .048*  [.003, .092] *t=*2.11, *p=*.035 |
| Slope after | -.136  [-.440, .167]  *t=*-.88, *p=*.379 | -.093  [-.515, .330] *t=*-.43, *p=*.668 | -.255  [-.799, .289] *t=*-.92, *p=*.358 | -.008*  [-.016, .000] *t=*-2.01, *p=*.044 | .002  [-.009, .013] *t=*.30, *p=*.767 | -.002  [-.013, .009] *t=*-.35, *p=*.725 |
| Slope change | -.208  [-.629, .213]  *t=*-.97, *p=*.332 | .158  [-.431, .746] *t=*.53, *p=*.599 | .154  [-.618, .925] *t=*.39, *p=*.696 | -.004  [-.015, .007] *t=*-.64, *p=*.520 | .000  [-.016, .015] *t=*-.03, *p=*.972 | .005  [-.011, .021] *t=*.61, *p=*.539 |
| *R^2^* | .173 | .177 | .162 | .243 | .220 | .232 |
| Adjusted *R^2^* | .159 | .163 | .148 | .230 | .207 | .219 |
| Observations | 424,969 | 301,380 | 301,380 | 424,969 | 301,380 | 301,380 |
| *df* | 418,115 | 296,544 | 296,544 | 418,115 | 296,544 | 296,544 |

*Notes: ***p<.001 **p<.01 *p<.05. We multiply political diversity by 100 to interpret the estimates as absolute percentage point changes. We normalize content diversity to z-scores (the number of standard deviations from the mean). All regressions control for user fixed effects and the number of tweets per day. The statistical significance of regression coefficients is tested using two-sided t-tests. Confidence intervals (95%) are provided in brackets, along with the corresponding t-statistics, degrees of freedom, and exact p-values. More details can be found in Methods: Interrupted Time Series (ITS) Analysis.*

**Supplementary Table 9. Political Stances of Taggers and Voters**

|  | **Individual tagging** | |
| --- | --- | --- |
|  | **Tags correcting left-leaning posters**  **(N=2,974)** | **Tags correcting right-leaning posters**  **(N=3,376)** |
| **Taggers’ political stance** | Left: 81.70%  Right: 18.30% | Left: 92.93%  Right: 7.07% |
|  | **Collective tagging** | |
|  | **Tags correcting left-leaning posters**  **(N=462)** | **Tags correcting right-leaning posters**  **(N=365)** |
| **Voters’ political stance** | Left: 66.56%  Right: 33.44% | Left: 86.22%  Right: 13.78% |

*Notes: Taggers are users who write individual tags (e.g., fact-checking replies), while voters are users who vote on the exposure of collective tags. For the methods used to measure the political stances of taggers and voters, refer to Supplementary Method 6: Political Stances of Taggers and Voters.*

**Supplementary Table 10. Comparing the Effects of Individual and Collective Tags When Left-leaning Taggers/Voters Correct Right-leaning Posters**

| **Outcome** | **Political diversity (%)** | | **Content diversity (z)** | |
| --- | --- | --- | --- | --- |
| **Controls** | **Initial results**  **(N = 424,969)** | **Left-leaning taggers/voters correcting right-leaning posters**  **(N = 241,681)** | **Initial results**  **(N = 424,969)** | **Left-leaning taggers/voters correcting right-leaning posters**  **(N = 241,681)** |
| Difference (Collective - Individual) |  |  |  |  |
| Slope before | .072  [-.219, .363]  *t=*.480, *p=*.628 | -.020  [-.431, .391]  *t=*-.09, *p=*.926 | -.005  [-.012, .003]  *t=*-1.17, *p=*.243 | -.003  [-.014, .008]  *t=*-.57, *p=*.568 |
| Immediate change | 1.279*  [.101, 2.457]  *t=*2.13, *p=*.033 | 1.780*  [.118, 3.441]  *t=*2.10, *p=*.036 | .070***  [.039, .102]  *t=*4.44, *p*<.001 | .076**  [.033, .119]  *t=*3.46, *p=*.001 |
| Slope after | -.136  [-.440, .167]  *t=*-.88, *p=*.379 | -.024  [-.450, .402]  *t=*-.11, *p=*.912 | -.008*  [-.016, .000]  *t=*-2.01, *p=*.044 | -.020***  [-.031, -.009]  *t=*-3.49, *p*<.001 |
| Slope change | -.208  [-.629, .213]  *t=*-.97, *p=*.332 | -.004  [-.597, .588]  *t=*-.01, *p=*.988 | -.004  [-.015, .007]  *t=*-.64, *p=*.520 | -.017*  [-.032, -.001]  *t=*-2.12, *p=*.034 |
| *R^2^* | .173 | .194 | .243 | .240 |
| Adjusted *R^2^* | .159 | .182 | .230 | .229 |
| Observations | 424,969 | 241,681 | 424,969 | 241,681 |
| *df* | 418,115 | 238,081 | 418,115 | 238,081 |

*Notes: ***p<.001 **p<.01 *p<.05. We multiply political diversity by 100 to interpret the estimates as absolute percentage point changes. We normalize content diversity to z-scores (the number of standard deviations from the mean). All regressions control for user fixed effects and the number of tweets per day. The statistical significance of regression coefficients is tested using two-sided t-tests. Confidence intervals (95%) are provided in brackets, along with the corresponding t-statistics, degrees of freedom, and exact p-values. More details can be found in Methods: Interrupted Time Series (ITS) Analysis.*

**Supplementary Table 11. Comparing the Effects of Individual and Collective Tags When Correcting Less Popular Posters**

| **Outcome** | **Political diversity (%)** | | **Content diversity (z)** | |
| --- | --- | --- | --- | --- |
| **Controls** | **Initial results**  **(N = 424,969)** | **Less popular posters**  **(N = 240,653)** | **Initial results**  **(N = 424,969)** | **Less popular posters**  **(N = 240,653)** |
| Difference (Collective - Individual) |  |  |  |  |
| Slope before | .072  [-.219, .363]  *t=*.480, *p=*.628 | -.584  [-1.298, .131]  *t=*-1.60, *p=*.110 | -.005  [-.012, .003]  *t=*-1.17, *p=*.243 | .007  [-.014, .027]  *t=*.64, *p=*.521 |
| Immediate change | 1.279*  [.101, 2.457]  *t=*2.13, *p=*.033 | 3.612*  [.824, 6.399]  *t=*2.54, *p=*.011 | .070***  [.039, .102]  *t=*4.44, *p*<.001 | .081*  [.000, .162]  *t=*1.97, *p=*.049 |
| Slope after | -.136  [-.440, .167]  *t=*-.88, *p=*.379 | .056  [-.658, .769]  *t=*.15, *p=*.878 | -.008*  [-.016, .000]  *t=*-2.01, *p=*.044 | -.005  [-.026, .016]  *t=*-.48, *p=*.629 |
| Slope change | -.208  [-.629, .213]  *t=*-.97, *p=*.332 | .640  [-.372, 1.651]  *t=*1.24, *p=*.215 | -.004  [-.015, .007]  *t=*-.64, *p=*.520 | -.012  [-.041, .017]  *t=*-.79, *p=*.427 |
| *R^2^* | .173 | .172 | .243 | .244 |
| Adjusted *R^2^* | .159 | .155 | .230 | .228 |
| Observations | 424,969 | 240,653 | 424,969 | 240,653 |
| *df* | 418,115 | 235,632 | 418,115 | 235,632 |

*Notes: ***p<.001 **p<.01 *p<.05. We multiply political diversity by 100 to interpret the estimates as absolute percentage point changes. We normalize content diversity to z-scores (the number of standard deviations from the mean). All regressions control for user fixed effects and the number of tweets per day. The statistical significance of regression coefficients is tested using two-sided t-tests. Confidence intervals (95%) are provided in brackets, along with the corresponding t-statistics, degrees of freedom, and exact p-values. More details can be found in Methods: Interrupted Time Series (ITS) Analysis.*

**Supplementary Table 12. Estimates of Interrupted Time Series (ITS) Models.**

| Type of misinformation tagging | Individual | Collective | Difference  (Collective - Individual) |
| --- | --- | --- | --- |
| Slope before posting the tweet | *β_1_* | *β_1_+β_4_* | *β_4_* |
| Immediate intercept change after misinformation tagging | *β_2_* | *β_2_+β_5_* | *β_5_* |
| Slope after misinformation tagging | *β_1_+β_3_* | *β_1_+β_3_+β_4_+β_6_* | *β_4_+β_6_* |
| Slope change (after - before) | *β_3_* | *β_3_+β_6_* | *β_6_* |

**Supplementary Table 13. Estimates of Delayed Feedback (DF) Models.**

| Type of misinformation tagging | Individual | Collective | Difference (Collective - Individual) |
| --- | --- | --- | --- |
| Difference in Pre-Post Change (Treatment - Control) | *β_1_* | *β_1_+β_2_* | *β_2_* |

**Supplementary Table 14. Slope Differences between Control and Treatment Group**

| **Outcome** | **Political diversity (%)** | **Content diversity (z)** |
| --- | --- | --- |
| Day | .313  [-.525, 1.151]  *t*(10)=.833, *p=*.424 | -.002  [-.035, .031]  *t*(10)=-.122, *p=*.905 |
| Treatment Group | -.620  [-5.918, 4.678]  *t*(10)=-.261, *p=*.799 | .102  [-.109, .312]  *t*(10)=1.075, *p=*.307 |
| Day × Treatment Group | -.493  [-1.644, .725]  *t*(10)=-.864, *p=*.408 | .011  [-0.036, .058] *t*(10)=.511, *p=*.620 |
| *R^2^* | .382 | .548 |
| Adjusted *R^2^* | .197 | .412 |
| Observations | 14 | 14 |

*Notes: ***p<.001 **p<.01 *p<.05. We multiply political diversity by 100 to interpret the estimates as absolute percentage point changes. We normalize content diversity to z-scores (the number of standard deviations from the mean). All regressions control for user fixed effects and the number of tweets per day. The statistical significance of regression coefficients is tested using two-sided t-tests. Confidence intervals (95%) are provided in brackets, along with the corresponding t-statistics, degrees of freedom, and exact p-values.*

**Supplementary Table 15. Robustness Checks for Interrupted Time Series (ITS) Models on Political Diversity**

| **Outcome** | **Political diversity (%)** | | | |
| --- | --- | --- | --- | --- |
| **Robustness checks** | **Initial results**  **(*N*=424,969)** | **Removing bots**  **(N=401,188)** | **Controlling credibility**  **(N=424,969)** | **Without negative sentiments**  **(N=268,626)** |
| Individual |  |  |  |  |
| Slope before | .237***  [.125, .349]  *t=*4.14, *p*<.001 | .221***  [.108, .335]  *t=*3.81, *p*<.001 | .229***  [.121, .338]  *t=*4.14, p<.001 | .224**  [.079, .369]  *t=*3.03, *p=*.002 |
| Immediate change | -1.009***  [-1.447, -.571]  *t=*-4.52, *p*<.001 | -.882***  [-1.326, -.438]  *t=*-3.89, *p*<.001 | -.972***  [-1.395, -.549]  *t=*-4.50, p<.001 | -1.470***  [-2.032, -.909]  *t=*-5.13, p<.001 |
| Slope after | .087  [-.020, .194]  *t=*1.60, *p=*.110 | .073  [-.035, .181]  *t=*1.32, *p=*.185 | .035  [-.068, .138]  *t=*.66, *p=*.509 | .237**  [.100, .374]  *t=*3.39, *p=*.001 |
| Slope change | -.150  [-.306, .006]  *t=*-1.89, *p=*.059 | -.148  [-.306, .010]  *t=*-1.84, *p=*.066 | -.194*  [-.345, -.044]  *t=*-2.53, *p=*.011 | .013  [-.188, .214]  *t=*.13, *p=*.899 |
| Collective |  |  |  |  |
| Slope before | .309*  [.041, .578]  *t=*2.26, *p=*.024 | .339*  [.045, .634]  *t=*2.26, *p=*.024 | .225  [-.034, .485]  *t=*1.70, *p=*.089 | .336  [-.011, .684]  *t=*1.90, *p=*.058 |
| Immediate change | .270  [-.824, 1.363]  *t=*.48, *p=*.629 | .223  [-.992, 1.438]  *t=*.36, *p=*.719 | .389  [-.667, 1.446]  *t=*.72, *p=*.470 | .375  [-1.040, 1.790]  *t=*.52, *p=*.604 |
| Slope after | -.049  [-.334, .235]  *t=*-.34, *p=*.734 | -.117  [-.434, .200]  *t=*-.72, *p=*.470 | -.023  [-.298, .252]  *t=*-.16, *p=*.869 | -.103  [-.473, .268]  *t=*-.54, *p=*.587 |
| Slope change | -.358  [-.749, .033]  *t=*-1.80, *p=*.072 | -.456*  [-.888, -.024]  *t=*-2.07, *p=*.039 | -.248  [-.626, .129]  *t=*-1.29, *p=*.198 | -.439  [-.947, .069]  *t=*-1.69, *p=*.090 |
| Difference (Collective - Individual) |  |  |  |  |
| Slope before | .072  [-.219, .363]  *t=*.48, *p=*.628 | .118  [-.198, .434]  *t=*.73, *p=*.464 | -.004  [-.285, .277]  *t=*-.03, *p=*.978 | .112  [-.264, .489]  *t=*.58, *p=*.559 |
| Immediate change | 1.279*  [.101, 2.457]  *t=*2.13, *p=*.033 | 1.105  [-.188, 2.399]  *t=*1.67, *p=*.094 | 1.361*  [.223, 2.499]  *t=*2.34, *p=*.019 | 1.845*  [.323, 3.368]  *t=*2.38, *p=*.018 |
| Slope after | -.136  [-.440, .167]  *t=*-.88, *p=*.379 | -.190  [-.525, .145]  *t=*-1.11, *p=*.266 | -.058  [-.351, .236]  *t=*-.39, *p=*.699 | -.340  [-.735, .056]  *t=*-1.68, *p=*.092 |
| Slope change | -.208  [-.629, .213]  *t=*-.97, *p=*.332 | -.308  [-.768, .152]  *t=*-1.31, *p=*.190 | -.054  [-.461, .353]  *t=*-.26, *p=*.795 | -.452  [-.998, .094]  *t=*-1.62, *p=*.105 |
| *R^2^* | .173 | .175 | .227 | .182 |
| Adjusted *R^2^* | .159 | .162 | .215 | .162 |
| Observations | 424,969 | 401,188 | 424,969 | 268,626 |
| *df* | 418,115 | 394,654 | 418,114 | 262,015 |

*Notes: ***p<.001 **p<.01 *p<.05. We multiply political diversity by 100 to interpret the estimates as absolute percentage point changes. We normalize content diversity to z-scores (the number of standard deviations from the mean). All regressions control for user fixed effects and the number of tweets per day. The statistical significance of regression coefficients is tested using two-sided t-tests. Confidence intervals (95%) are provided in brackets, along with the corresponding t-statistics, degrees of freedom, and exact p-values. More details can be found in Methods: Interrupted Time Series (ITS) Analysis.*

**Supplementary Table 16. Robustness Checks for Interrupted Time Series (ITS) Models on Content Diversity**

| **Outcome** | **Content diversity (*z*)** | | | |
| --- | --- | --- | --- | --- |
| **Robustness checks** | **Initial results**  **(*N*=424,969)** | **Removing bots**  **(N=401,188)** | **Controlling credibility**  **(N=424,969)** | **Without negative sentiments**  **(N=268,626)** |
| Individual |  |  |  |  |
| Slope before | .007***  [.004, .010]  *t=*4.79, *p*<.001 | .006***  [.003, .009]  *t=*3.94, p<.001 | .007***  [.004, .010]  *t=*4.78, p<.001 | .007**  [.003, .011]  *t=*3.19, *p=*.001 |
| Immediate change | -.030***  [-.042, -.019]  *t=*-5.10, *p*<.001 | -.026***  [-.037, -.014]  *t=*-4.27, p<.001 | -.030***  [-.042, -.018]  *t=*-5.08, p<.001 | -.027**  [-.042, -.011]  *t=*-3.35, *p=*.001 |
| Slope after | -.003  [-.006, .000]  *t=*-1.95, *p=*.051 | -.003*  [-.006, .000]  *t=*-2.25, *p=*.024 | -.003*  [-.006, .000]  *t=*-2.02, *p=*.043 | -.005*  [-.009, -.001]  *t=*-2.46, *p=*.014 |
| Slope change | -.010***  [-.014, -.006]  *t=*-4.79, *p*<.001 | -.009***  [-.013, -.005]  *t=*-4.38, p<.001 | -.010***  [-.014, -.006]  *t=*-4.83, p<.001 | -.011***  [-.017, -.006]  *t=*-3.99, p<.001 |
| Collective |  |  |  |  |
| Slope before | .003  [-.004, .010]  *t=*.74, *p=*.461 | .001  [-.007, .009]  *t=*.32, *p=*.749 | .003  [-.005, .010]  *t=*.69, *p=*.488 | .012*  [.003, .022]  *t=*2.47, *p=*.014 |
| Immediate change | .040**  [.012, .069]  *t=*2.74, *p=*.006 | .052**  [.020, .084]  *t=*3.16, *p=*.002 | .041**  [.012, .070]  *t=*2.76, *p=*.006 | .009  [-.031, .048]  *t=*.43, *p=*.666 |
| Slope after | -.011**  [-.019, -.004]  *t=*-2.88, *p=*.004 | -.013**  [-.022, -.005]  *t=*-3.15, *p=*.002 | -.011**  [-.018, -.003]  *t=*-2.87, *p=*.004 | -.006  [-.016, .004]  *t=*-1.12, *p=*.262 |
| Slope change | -.014**  [-.024, -.003]  *t=*-2.60, *p=*.009 | -.015*  [-.026, -.003]  *t=*-2.52, *p=*.012 | -.013*  [-.024, -.003]  *t=*-2.56, *p=*.010 | -.018*  [-.032, -.004]  *t=*-2.51, *p=*.012 |
| Difference (Collective - Individual) |  |  |  |  |
| Slope before | -.005  [-.012, .003]  *t=*-1.17, *p=*.243 | -.005  [-.013, .004]  *t=*-1.12, *p=*.262 | -.005  [-.012, .003]  *t=*-1.20, *p=*.228 | .006  [-.005, .016]  *t=*1.05, *p=*.293 |
| Immediate change | .070***  [.039, .102]  *t=*4.44, *p*<.001 | .077***  [.043, .111]  *t=*4.43, p<.001 | .071***  [.040, .102]  *t=*4.45, p<.001 | .035  [-.007, .078]  *t=*1.63, *p=*.102 |
| Slope after | -.008*  [-.016, .000]  *t=*-2.01, *p=*.044 | -.010*  [-.019, -.001]  *t=*-2.25, *p=*.025 | -.008*  [-.016, .000]  *t=*-1.98, *p=*.048 | -.001  [-.012, .010]  *t=*-.20, *p=*.843 |
| Slope change | -.004  [-.015, .007]  *t=*-.64, *p=*.520 | -.005  [-.018, .007]  *t=*-.87, *p=*.386 | -.003  [-.014, .008]  *t=*-.59, *p=*.554 | -.007  [-.022, .008]  *t=*-.87, *p=*.385 |
| *R^2^* | .243 | .240 | .243 | .319 |
| Adjusted *R^2^* | .230 | .227 | .231 | .302 |
| Observations | 424,969 | 401,188 | 424,969 | 268,626 |
| *df* | 418,115 | 394,654 | 418,114 | 262,015 |

*Notes: ***p<.001 **p<.01 *p<.05. We multiply political diversity by 100 to interpret the estimates as absolute percentage point changes. We normalize content diversity to z-scores (the number of standard deviations from the mean). All regressions control for user fixed effects and the number of tweets per day. The statistical significance of regression coefficients is tested using two-sided t-tests. Confidence intervals (95%) are provided in brackets, along with the corresponding t-statistics, degrees of freedom, and exact p-values. More details can be found in Methods: Interrupted Time Series (ITS) Analysis.*

**Supplementary Table 17. Robustness Checks for Delayed Feedback (DF) Models**

| **Outcome** | **Political diversity (%)** | | | |
| --- | --- | --- | --- | --- |
| **Robustness checks** | **Initial results**  **(*N*=8,901)** | **Removing bots**  **(*N*=8,442)** | **Controlling credibility**  **(*N*=8,901)** | **Without negative sentiments**  **(*N*=5,615)** |
| Individual | -5.886**  [-9.633, -2.138] *t=*-3.08, *p=*.002 | -5.348**  [-9.154, -1.542]  *t=*-2.75, *p=*.006 | -4.958**  [-8.591, -1.324]  *t=*-2.67, *p=*.007 | -8.075**  [-12.757, -3.394]  *t=*-3.38, *p=*.001 |
| Collective | 1.219  [-4.777, 7.215]  *t=*.40, *p=*.690 | -.899  [-7.493, 5.694]  *t=*-.27, *p=*.789 | 2.246  [-3.568, 8.060]  *t=*.76, *p=*.449 | 3.178  [-4.416, 10.773]  *t=*.82, *p=*.412 |
| Difference (Collective - Individual) | 7.105*  [.069, 14.140]  *t=*1.98, *p=*.048 | 4.449  [-3.127, 12.025]  *t=*1.15, *p=*.250 | 7.204*  [.383, 14.025]  *t=*2.07, *p=*.038 | 11.253*  [2.361, 20.146]  *t=*2.48, *p=*.013 |
| *R^2^* | .274 | .272 | .318 | .349 |
| Adjusted *R^2^* | .211 | .208 | .258 | .265 |
| Observations | 8,901 | 8,442 | 8,901 | 5,615 |
| *df* | 8,182 | 7,766 | 8,181 | 4,973 |
| **Outcome** | **Content diversity (z)** | | | |
| **Robustness checks** | **Initial results**  **(*N*=8,901)** | **Removing bots**  **(*N*=8,442)** | **Controlling credibility**  **(*N*=8,901)** | **Without negative sentiments**  **(*N*=5,615)** |
| Individual | .018  [-.062, .099]  *t=*.45, *p=*.652 | .026  [-.057, .108]  *t=*.61, *p=*.543 | .022  [-.058, .103]  *t=*.55, *p=*.584 | .043  [-.068, .154]  *t=*.76, *p=*.447 |
| Collective | .274***  [.145, .403]  *t=*4.17, *p*<.001 | .292***  [.149, .435]  *t=*4.00, p<.001 | .279***  [.150, .407]  *t=*4.25, p<.001 | .294**  [.114, .474]  *t=*3.20, *p=*.001 |
| Difference (Collective - Individual) | .256**  [.105, .407]  *t=*3.32, *p=*.001 | .266**  [.102, .431]  *t=*3.18, *p=*.001 | .256**  [.105, .407]  *t=*3.33, *p=*.001 | .251*  [.040, .462]  *t=*2.33, *p=*.020 |
| *R^2^* | .358 | .349 | .359 | .428 |
| Adjusted *R^2^* | .301 | .293 | .303 | .354 |
| Observations | 8,901 | 8,442 | 8,901 | 5,615 |
| *df* | 8,182 | 7,766 | 8,181 | 4,973 |

*Notes: ***p<.001 **p<.01 *p<.05. Each cell presents the difference in pre-post change (treatment group - control group) in each outcome. We multiply political diversity by 100 to interpret the estimates as absolute percentage point changes. We normalize content diversity to z-scores (the number of standard deviations from the mean). All regressions control for user fixed effects and the number of tweets per day. The statistical significance of regression coefficients is tested using two-sided t-tests. Confidence intervals (95%) are provided in brackets, along with the corresponding t-statistics, degrees of freedom, and exact p-values. More details can be found in Methods: Delayed Feedback (DF) Analysis.*

**Supplementary Table 18. Interrupted Times Series (ITS) Models after Removing Tweets Mentioning the Experience of Receiving Tags or Interacting with Taggers**

| **Outcome** | **Political Diversity (%)** | | **Content Diversity (z)** | |
| --- | --- | --- | --- | --- |
| **Robustness checks** | **Initial results**  **(*N*=424,969)** | **Removing Tweets Mentioning Tags or Replying to Taggers**  **(N=422,991)** | **Controlling credibility**  **(N=424,969)** | **Removing Tweets Mentioning Tags or Replying to Taggers**  **(N=422,991)** |
| Individual |  |  |  |  |
| Slope before | .237***  [.125, .349]  *t=*4.14, p<.001 | .228***  [.116, .341]  *t=*3.97, p<.001 | .007***  [.004, .010] *t=*4.79, *p*<.001 | .007***  [.004, .010]  *t=*4.62, p<.001 |
| Immediate change | -1.009***  [-1.447, -.571]  *t=*-4.52, p<.001 | -1.048***  [-1.488, -.608]  *t=*-4.67, p<.001 | -.030***  [-.042, -.019]  *t=*-5.10, *p*<.001 | -.030***  [-.041, -.018]  *t=*-5.00, p<.001 |
| Slope after | .087  [-.020, .194]  *t=*1.60, *p=*.110 | .107*  [.000, .215]  *t=*1.97, *p=*.049 | -.003  [-.006, .000]  *t=*-1.95, *p=*.051 | -.003  [-.006, .000]  *t=*-1.88, *p=*.060 |
| Slope change | -.150  [-.306, .006]  *t=*-1.89, *p=*.059 | -.121  [-.277, .036]  *t=*-1.51, *p=*.130 | -.010***  [-.014, -.006]  *t=*-4.79, *p*<.001 | -.010***  [-.014, -.006]  *t=*-4.62, p<.001 |
| Collective |  |  |  |  |
| Slope before | .309*  [.041, .578]  *t=*2.26, *p=*.024 | .307*  [.038, .575]  *t=*2.24, *p=*.025 | .003  [-.004, .010]  *t=*.74, *p=*.461 | .003  [-.004, .010]  *t=*.73, *p=*.464 |
| Immediate change | .270  [-.824, 1.363]  *t=*.48, *p=*.629 | .262  [-.830, 1.354]  *t=*.47, *p=*.638 | .040**  [.012, .069]  *t=*2.74, *p=*.006 | .041**  [.012, .069]  *t=*2.75, *p=*.006 |
| Slope after | -.049  [-.334, .235]  *t=*-.34, *p=*.734 | -.046  [-.330, .238]  *t=*-.32, *p=*.752 | -.011**  [-.019, -.004] *t=*-2.88, *p=*.004 | -.011**  [-.019, -.004]  *t=*-2.89, *p=*.004 |
| Slope change | -.358  [-.749, .033]  *t=*-1.80, *p=*.072 | -.352  [-.743, .038]  *t=*-1.77, *p=*.077 | -.014**  [-.024, -.003]  *t=*-2.60, *p=*.009 | -.014**  [-.024, -.003]  *t=*-2.61, *p=*.009 |
| Difference (Collective - Individual) |  |  |  |  |
| Slope before | .072  [-.219, .363]  *t=*.48, *p=*.628 | .078  [-.212, .369]  *t=*.53, *p=*.597 | -.005  [-.012, .003]  *t=*-1.17, *p=*.243 | -.004  [-.012, .003]  *t=*-1.11, *p=*.266 |
| Immediate change | 1.279*  [.101, 2.457]  *t=*2.13, *p=*.033 | 1.310*  [.133, 2.487]  *t=*2.18, *p=*.029 | .070***  [.039, .102]  *t=*4.44, *p*<.001 | .070***  [.039, .101]  *t=*4.42, p<.001 |
| Slope after | -.136  [-.440, .167]  *t=*-.88, *p=*.379 | -.153  [-.457, .150]  *t=*-.99, *p=*.322 | -.008*  [-.016, .000]  *t=*-2.01, *p=*.044 | -.008*  [-.016, .000]  *t=*-2.04, *p=*.041 |
| Slope change | -.208  [-.629, .213]  *t=*-.97, *p=*.332 | -.232  [-.652, .189]  *t=*-1.08, *p=*.280 | -.004  [-.015, .007]  *t=*-.64, *p=*.520 | -.004  [-.015, .007]  *t=*-.70, *p=*.481 |
| *R^2^* | .173 | .172 | .243 | .243 |
| Adjusted *R^2^* | .159 | .159 | .230 | .230 |
| Observations | 424,969 | 422,991 | 424,969 | 422,991 |
| *df* | 418,115 | 416,183 | 418,115 | 416,183 |

*Notes: ***p<.001 **p<.01 *p<.05. We multiply political diversity by 100 to interpret the estimates as absolute percentage point changes. We normalize content diversity to z-scores (the number of standard deviations from the mean). All regressions control for user fixed effects and the number of tweets per day. The statistical significance of regression coefficients is tested using two-sided t-tests. Confidence intervals (95%) are provided in brackets, along with the corresponding t-statistics, degrees of freedom, and exact p-values. More details can be found in Methods: Interrupted Time Series (ITS) Analysis.*

**Supplementary Table 19. Delayed Feedback (DF) Models after Removing Tweets Mentioning the Experience of Receiving Tags or Interacting with Taggers**

| **Outcome** | **Political diversity (%)** | | **Content diversity (z)** | |
| --- | --- | --- | --- | --- |
| **Robustness checks** | **Initial results**  **(*N*=8,901)** | **Removing Tweets Mentioning Tags or Replying to Taggers**  **(N=8,787)** | **Initial results**  **(*N*=8,901)** | **Removing Tweets Mentioning Tags or Replying to Taggers**  **(N=8,787)** |
| Individual | -5.886**  [-9.633, -2.138] *t=*-3.08, *p=*.002 | -5.329**  [-9.139, -1.519]  *t=*-2.74, *p=*.006 | .018  [-.062, .099]  *t=*.45, *p=*.652 | .016  [-.066, .098]  *t=*.38, *p=*.704 |
| Collective | 1.219  [-4.777, 7.215]  *t=*.40, *p=*.690 | 1.070  [-4.914, 7.055]  *t=*.35, *p=*.726 | .274***  [.145, .403]  *t=*4.17, *p*<.001 | .276***  [.148, .405]  *t=*4.22, p<.001 |
| Difference (Collective - Individual) | 7.105*  [.069, 14.140]  *t=*1.98, *p=*.048 | 6.400  [-.659, 13.458]  *t=*1.78, *p=*.076 | .256**  [.105, .407]  *t=*3.32, *p=*.001 | .260**  [.109, .412]  *t=*3.37, *p=*.001 |
| *R^2^* | .274 | .277 | .358 | .360 |
| Adjusted *R^2^* | .211 | .214 | .301 | .305 |
| Observations | 8,901 | 8,787 | 8,901 | 8,787 |
| *df* | 8,182 | 8,088 | 8,182 | 8,088 |

*Notes: ***p<.001 **p<.01 *p<.05. Each cell presents the difference in pre-post change (treatment group - control group) in each outcome. We multiply political diversity by 100 to interpret the estimates as absolute percentage point changes. We normalize content diversity to z-scores (the number of standard deviations from the mean). All regressions control for user fixed effects and the number of tweets per day. The statistical significance of regression coefficients is tested using two-sided t-tests. Confidence intervals (95%) are provided in brackets, along with the corresponding t-statistics, degrees of freedom, and exact p-values. More details can be found in Methods: Delayed Feedback (DF) Analysis.*

**Supplementary Table 20. Interrupted Times Series (ITS) Models after Limiting the Sample to Corrective Individual Tags**

| **Outcome** | **Political diversity (%)** | | **Content diversity (z)** | |
| --- | --- | --- | --- | --- |
| **Robustness checks** | **Initial results**  **(N = 424,969)** | **Limiting the sample**  **(N = 369,070)** | **Initial results**  **(N = 424,969)** | **Limiting the sample**  **(N = 369,070)** |
| Individual |  |  |  |  |
| Slope before | .237***  [.125, .349]  *t=*4.14, p<.001 | .284***  [.164, .405]  *t=*4.62, p<.001 | .007***  [.004, .010] *t=*4.79, *p*<.001 | .009***  [.006, .012]  *t=*5.46, p<.001 |
| Immediate change | -1.009***  [-1.447, -.571]  *t=*-4.52, p<.001 | -1.086***  [-1.558, -.614]  *t=*-4.51, p<.001 | -.030***  [-.042, -.019]  *t=*-5.10, *p*<.001 | -.039***  [-.051, -.026]  *t=*-6.11, p<.001 |
| Slope after | .087  [-.020, .194]  *t=*1.60, *p=*.110 | .070  [-.045, .185]  *t=*1.19, *p=*.232 | -.003  [-.006, .000]  *t=*-1.95, *p=*.051 | -.003*  [-.007, .000]  *t=*-2.24, *p=*.025 |
| Slope change | -.150  [-.306, .006]  *t=*-1.89, *p=*.059 | -.214*  [-.382, -.046]  *t=*-2.50, *p=*.012 | -.010***  [-.014, -.006]  *t=*-4.79, *p*<.001 | -.012***  [-.017, -.008]  *t=*-5.46, p<.001 |
| Collective |  |  |  |  |
| Slope before | .309*  [.041, .578]  *t=*2.26, *p=*.024 | .309*  [.042, .576]  *t=*2.27, *p=*.023 | .003  [-.004, .010]  *t=*.74, *p=*.461 | .003  [-.004, .010]  *t=*.75, *p=*.454 |
| Immediate change | .270  [-.824, 1.363]  *t=*.48, *p=*.629 | .270  [-.816, 1.355]  *t=*.49, *p=*.626 | .040**  [.012, .069]  *t=*2.74, *p=*.006 | .040**  [.012, .069]  *t=*2.76, *p=*.006 |
| Slope after | -.049  [-.334, .235]  *t=*-.34, *p=*.734 | -.049  [-.332, .233]  *t=*-.34, *p=*.732 | -.011**  [-.019, -.004]  *t=*-2.88, *p=*.004 | -.011**  [-.018, -.004]  *t=*-2.90, *p=*.004 |
| Slope change | -.358  [-.749, .033]  *t=*-1.80, *p=*.072 | -.358  [-.746, .030]  *t=*-1.81, *p=*.070 | -.014**  [-.024, -.003]  *t=*-2.60, *p=*.009 | -.014**  [-.024, -.003]  *t=*-2.63, *p=*.009 |
| Individual vs. collective |  |  |  |  |
| Slope before | .072  [-.219, .363]  *t=*.48, *p=*.628 | .025  [-.268, .317]  *t=*.17, *p=*.869 | -.005  [-.012, .003]  *t=*-1.17, *p=*.243 | -.006  [-.014, .002]  *t=*-1.57, *p=*.117 |
| Immediate change | 1.279*  [.101, 2.457]  *t=*2.13, *p=*.033 | 1.356*  [.172, 2.539]  *t=*2.24, *p=*.025 | .070***  [.039, .102]  *t=*4.44, *p*<.001 | .079***  [.048, .111]  *t=*4.97, p<.001 |
| Slope after | -.136  [-.440, .167]  *t=*-.88, *p=*.379 | -.120  [-.424, .185]  *t=*-.77, *p=*.442 | -.008*  [-.016, .000]  *t=*-2.01, *p=*.044 | -.008  [-.016, .000]  *t=*-1.84, *p=*.066 |
| Slope change | -.208  [-.629, .213]  *t=*-.97, *p=*.332 | -.144  [-.567, .279]  *t=*-.67, *p=*.504 | -.004  [-.015, .007]  *t=*-.64, *p=*.520 | -.001  [-.013, .010]  *t=*-.24, *p=*.808 |
| *R^2^* | .173 | .174 | .243 | .242 |
| Adjusted *R^2^* | .159 | .161 | .230 | .230 |
| Observations | 424,969 | 369,070 | 424,969 | 369,070 |
| *df* | 418,115 | 363,273 | 418,115 | 363,273 |

*Notes: ***p<.001 **p<.01 *p<.05. We multiply political diversity by 100 to interpret the estimates as absolute percentage point changes. We normalize content diversity to z-scores (the number of standard deviations from the mean). All regressions control for user fixed effects and the number of tweets per day. The statistical significance of regression coefficients is tested using two-sided t-tests. Confidence intervals (95%) are provided in brackets, along with the corresponding t-statistics, degrees of freedom, and exact p-values. More details can be found in Methods: Interrupted Time Series (ITS) Analysis.*

**Supplementary Table 21. Delayed Feedback (DF) Models after Limiting the Sample to Corrective Individual Tags**

| **Outcome** | **Political diversity (%)** | | **Content diversity (z)** | |
| --- | --- | --- | --- | --- |
| **Robustness checks** | **Initial results**  **(*N*=8,901)** | **Limiting the sample**  **(N=8,145)** | **Initial results**  **(*N*=8,901)** | **Limiting the sample**  **(N=8,145)** |
| Individual | -5.886**  [-9.633, -2.138]  *t=*-3.08, *p=*.002 | -7.135***  [-11.068, -3.203] *t=*-3.56, p<.001 | .018  [-.062, .099]  *t=*.45, *p=*.652 | .026  [-.061, .112]  *t=*.59, *p=*.557 |
| Collective | 1.219  [-4.777, 7.215]  *t=*.40, *p=*.690 | 1.336  [-4.532, 7.204]  *t=*.45, *p=*.655 | .274***  [.145, .403]  *t=*4.17, *p*<.001 | .272***  [.143, .401]  *t=*4.13, p<.001 |
| Difference (Collective - Individual) | 7.105*  [.069, 14.140]  *t=*1.98, *p=*.048 | 8.471*  [1.445, 15.498]  *t=*2.36, *p=*.018 | .256**  [.105, .407]  *t=*3.32, *p=*.001 | .246**  [.091, .400]  *t=*3.12, *p=*.002 |
| *R^2^* | .274 | .274 | .358 | .367 |
| Adjusted *R^2^* | .211 | .213 | .301 | .313 |
| Observations | 8,901 | 8,145 | 8,901 | 8,145 |
| *df* | 8,182 | 7,508 | 8,182 | 7,508 |

*Notes: ***p<.001 **p<.01 *p<.05. Each cell presents the difference in pre-post change (treatment group - control group) in each outcome. We multiply political diversity by 100 to interpret the estimates as absolute percentage point changes. We normalize content diversity to z-scores (the number of standard deviations from the mean). All regressions control for user fixed effects and the number of tweets per day. The statistical significance of regression coefficients is tested using two-sided t-tests. Confidence intervals (95%) are provided in brackets, along with the corresponding t-statistics, degrees of freedom, and exact p-values. More details can be found in Methods: Delayed Feedback (DF) Analysis.*

**Supplementary Table 22. Comparing the Effects of Individual and Collective Tagging after Removing Non-Responders**

| **Outcome** | **Political diversity (%)** | | **Content diversity (z)** | |
| --- | --- | --- | --- | --- |
| **Robustness checks** | **Initial results**  **(N = 424,969)** | **Removing non-responders**  **(N = 369,070)** | **Initial results**  **(N = 424,969)** | **Removing non-responders**  **(N = 369,070)** |
| Individual |  |  |  |  |
| Slope before | .237***  [.125, .349]  *t=*4.14, p<.001 | .514***  [.290, .737]  *t=*4.51, p<.001 | .007***  [.004, .010]  *t=*4.79, *p*<.001 | .004  [-.002, .010]  *t=*1.41, *p=*.159 |
| Immediate change | -1.009***  [-1.447, -.571]  *t=*-4.52, p<.001 | -1.481**  [-2.337, -.626]  *t=*-3.39, *p=*.001 | -.030***  [-.042, -.019]  *t=*-5.10, *p*<.001 | -.026*  [-.048, -.004]  *t=*-2.30, *p=*.021 |
| Slope after | .087  [-.020, .194]  *t=*1.60, *p=*.110 | .145  [-.063, .354]  *t=*1.37, *p=*.172 | -.003  [-.006, .000]  *t=*-1.95, *p=*.051 | -.005  [-.010, .000]  *t=*-1.81, *p=*.071 |
| Slope change | -.150  [-.306, .006]  *t=*-1.89, *p=*.059 | -.368*  [-.676, -.061]  *t=*-2.35, *p=*.019 | -.010***  [-.014, -.006]  *t=*-4.79, *p*<.001 | -.009*  [-.017, -.001]  *t=*-2.25, *p=*.025 |
| Collective |  |  |  |  |
| Slope before | .309*  [.041, .578]  *t=*2.26, *p=*.024 | .311*  [.022, .600]  *t=*2.11, *p=*.035 | .003  [-.004, .010]  *t=*.74, *p=*.461 | .003  [-.005, .010]  *t=*.71, *p=*.475 |
| Immediate change | .270  [-.824, 1.363]  *t=*.48, *p=*.629 | .270  [-.905, 1.445]  *t=*.45, *p=*.652 | .040**  [.012, .069]  *t=*2.74, *p=*.006 | .040*  [.010, .071]  *t=*2.59, *p=*.010 |
| Slope after | -.049  [-.334, .235]  *t=*-.34, *p=*.734 | -.049  [-.355, .256]  *t=*-.32, *p=*.751 | -.011**  [-.019, -.004]  *t=*-2.88, *p=*.004 | -.011**  [-.019, -.003]  *t=*-2.72, *p=*.007 |
| Slope change | -.358  [-.749, .033]  *t=*-1.80, *p=*.072 | -.361  [-.781, .060]  *t=*-1.68, *p=*.093 | -.014**  [-.024, -.003]  *t=*-2.60, *p=*.009 | -.014*  [-.025, -.003]  *t=*-2.47, *p=*.014 |
| Individual vs. collective |  |  |  |  |
| Slope before | .072  [-.219, .363]  *t=*.48, *p=*.628 | -.203  [-.568, .162]  *t=*-1.09, *p=*.276 | -.005  [-.012, .003]  *t=*-1.17, *p=*.243 | -.001  [-.011, .008]  *t=*-.30, *p=*.766 |
| Immediate change | 1.279*  [.101, 2.457]  *t=*2.13, *p=*.033 | 1.752*  [.298, 3.205]  *t=*2.36, *p=*.018 | .070***  [.039, .102]  *t=*4.44, *p*<.001 | .067**  [.029, .105]  *t=*3.45, *p=*.001 |
| Slope after | -.136  [-.440, .167]  *t=*-.88, *p=*.379 | -.195  [-.565, .175]  *t=*-1.03, *p=*.302 | -.008*  [-.016, .000]  *t=*-2.01, *p=*.044 | -.006  [-.016, .004]  *t=*-1.23, *p=*.219 |
| Slope change | -.208  [-.629, .213]  *t=*-.97, *p=*.332 | .008  [-.513, .529]  *t=*.03, *p=*.976 | -.004  [-.015, .007]  *t=*-.64, *p=*.520 | -.005  [-.018, .009]  *t=*-.67, *p=*.506 |
| *R^2^* | .173 | .162 | .243 | .243 |
| Adjusted *R^2^* | .159 | .147 | .230 | .229 |
| Observations | 424,969 | 167,766 | 424,969 | 167,766 |
| *df* | 418,115 | 164,724 | 418,115 | 164,724 |

*Notes: ***p<.001 **p<.01 *p<.05. We multiply political diversity by 100 to interpret the estimates as absolute percentage point changes. We normalize content diversity to z-scores (the number of standard deviations from the mean). All regressions control for user fixed effects and the number of tweets per day. The statistical significance of regression coefficients is tested using two-sided t-tests. Confidence intervals (95%) are provided in brackets, along with the corresponding t-statistics, degrees of freedom, and exact p-values. More details can be found in Methods: Interrupted Time Series (ITS) Analysis.*

**Supplementary Table 23. Pairwise Differences in Content Diversity Before and After Collective Tagging**

| **A pair of weeks being compared** | **Difference in content diversity** |
| --- | --- |
| (Week 5) - (Week -5) | *β*=-.001, 95% CI=[-.033, .030], *t=*-.09, *p=*.929 |
| (Week 5) - (Week -4) | *β*=-.004, 95% CI=[-.032, .024], *t=*-.28, *p=*.776 |
| (Week 5) - (Week -3) | *β*=-.007, 95% CI=[-.033, .020], *t=*-.50, *p=*.615 |
| (Week 5) - (Week -2) | *β*=-.009, 95% CI=[-.036, .017], *t=*-.70, *p=*.482 |
| (Week 5) - (Week -1) | *β*=-.012, 95% CI=[-.040, .016], *t=*-.84, *p=*.398 |
| (Week 5) - (Week 0) | *β*=-.015, 95% CI=[-.046, .017], *t=*-.92, *p=*.357 |
| *R^2^* | .173 |
| Adjusted *R^2^* | .159 |
| Observations | 424,969 |
| *df* | 418,115 |

*Notes: ***p<.001 **p<.01 *p<.05.* *Difference in content diversity between the two weeks is identified using two-sided t-tests. Confidence intervals (95%) are provided in brackets, along with the corresponding t-statistics, degrees of freedom, and exact p-values. We multiply political diversity by 100 to interpret the estimates as absolute percentage point changes. We normalize content diversity to z-scores (the number of standard deviations from the mean).*

**Supplementary Table 24. Interrupted Time Series (ITS) Model Results for Political and Content Proximity to Taggers**

| **Outcome** | **Political Proximity (%)** | **Content Proximity (z)** |
| --- | --- | --- |
| Slope before | .146*  [.028, .264]  *t=*2.42, *p=*.015 | .003*  [.001, .006]  *t=*2.43, *p=*.015 |
| Immediate change | -.536*  [-.996, -.077]  *t=*-2.29, *p=*.022 | .007  [-.004, .018]  *t=*1.27, *p=*.206 |
| Slope after | -.057  [-.169, .055]  *t=*-.99, *p=*.320 | -.002  [-.004, .001]  *t=*-1.20, *p=*.230 |
| Slope change (After - Before) | -.203*  [-.366, -.039]  *t=*-2.43, *p=*.015 | -.005*  [-.009, -.001]  *t=*-2.57, *p=*.010 |
| *R^2^* | .555 | .437 |
| Adjusted *R^2^* | .547 | .427 |
| Observations | 317,442 | |
| *df* | 312,333 | |

*Notes: ***p<.001 **p<.01 *p<.05. We multiply political diversity by 100 to interpret the estimates as absolute percentage point changes. We normalize content diversity to z-scores (the number of standard deviations from the mean). All regressions control for user fixed effects and the number of tweets per day. The statistical significance of regression coefficients is tested using two-sided t-tests. Confidence intervals (95%) are provided in brackets, along with the corresponding t-statistics, degrees of freedom, and exact p-values. More details can be found in Methods: Interrupted Time Series (ITS) Analysis.*

**Supplementary Table 25. Effects of Major Events on Political and Content Diversity**

| **Outcome** | **Political diversity (%) (N = 424,969)** | **Content diversity (z) (N = 424,969)** |
| --- | --- | --- |
| Events |  |  |
| SARS-CoV-2 Omicron variant (November 24, 2021) | .152  [-.285, .588]  *t=*.68, *p=*.496 | .011  [-.001, .022]  *t=*1.83, *p=*.068 |
| Russian invasion of Ukraine (February 24, 2022) | 1.528***  [1.056, 1.999]  *t=*6.35, *p*<.001 | -.065***  [-.077, -.052]  *t=*-10.15, *p*<.001 |
| Acquisition of Twitter by Elon Musk (Initiated) (April 14, 2022) | 8.856*  [1.797, 15.916]  *t=*2.46, *p=*.014 | .410***  [.223, .597]  *t=*4.31, *p*<.001 |
| Acquisition of Twitter by Elon Musk (Completed) (October 27, 2022) | -.809  [-15.750, 14.131]  *t=*-.11, *p=*.915 | -.072  [-.468, .323]  *t=*-.36, *p=*.719 |
| 2022 United States elections (November 8, 2022) | 3.950  [-9.551, 17.450]  *t=*.57, *p=*.566 | .005  [-.352, .362]  *t=*.03, *p=*.979 |
| 2023 Turkey-Syria earthquakes (February 6, 2023) | .700  [-.269, 1.670]  *t=*1.42, *p=*.157 | .021  [-.005, .046]  *t=*1.57, *p=*.117 |
| *R^2^* | .172 | .240 |
| Adjusted *R^2^* | .159 | .228 |
| Observations | 424,969 | 424,969 |
| *df* | 418,115 | 418,116 |

*Notes: ***p<.001 **p<.01 *p<.05. We multiply political diversity by 100 to interpret the estimates as absolute percentage point changes. We normalize content diversity to z-scores (the number of standard deviations from the mean). All regressions control for user fixed effects and the number of tweets per day. The statistical significance of regression coefficients is tested using two-sided t-tests. Confidence intervals (95%) are provided in brackets, along with the corresponding t-statistics, degrees of freedom, and exact p-values.*

**Supplementary Table 26. Effects of Individual and Collective Tags after Controlling Major Events**

| **Outcome** | **Political diversity (%)** | | **Content diversity (z)** | |
| --- | --- | --- | --- | --- |
| **Adjustment** | **Initial results**  **(*N*=424,969)** | **Controlling Six Major Events (*N* = 424,969)** | **Initial results**  **(*N*=424,969)** | **Controlling Six Major Events (*N* = 424,969)** |
| Individual |  |  |  |  |
| Slope before | .237***  [.125, .349]  *t=*4.14, *p*<.001 | .208***  [.093, .322]  *t=*3.56, *p*<.001 | .007***  [.004, .010]  *t=*4.79, *p*<.001 | .007***  [.004, .011]  *t=*4.85, *p*<.001 |
| Immediate change | -1.009***  [-1.447, -.571]  *t=*-4.52, *p*<.001 | -1.106***  [-1.544, -.667]  *t=*-4.94, *p*<.001 | -.030***  [-.042, -.019]  *t=*-5.10, *p*<.001 | -.027***  [-.039, -.016]  *t=*-4.64, *p*<.001 |
| Slope after | .087  [-.020, .194]  *t=*1.60, *p=*.110 | -.052  [-.166, .062]  *t=*-.90, *p=*.369 | -.003  [-.006, .000]  *t=*-1.95, *p=*.051 | .000  [-.003, .003]  *t=*.31, *p=*.756 |
| Slope change | -.150  [-.306, .006]  *t=*-1.89, *p=*.059 | -.260**  [-.419, -.102]  *t=*-3.22, *p=*.001 | -.010***  [-.014, -.006]  *t=*-4.79, *p*<.001 | -.007**  [-.011, -.003]  *t=*-3.28, *p=*.001 |
| Collective |  |  |  |  |
| Slope before | .309*  [.041, .578]  *t=*2.26, *p=*.024 | .332*  [.051, .612]  *t=*2.32, *p=*.020 | .003  [-.004, .010]  *t=*.74, *p=*.461 | .002  [-.005, .010]  *t=*.62, *p=*.536 |
| Immediate change | .270  [-.824, 1.363]  *t=*.48, *p=*.629 | .258  [-.836, 1.351]  *t=*.46, *p=*.644 | .040**  [.012, .069]  *t=*2.74, *p=*.006 | .040**  [.011, .069]  *t=*2.70, *p=*.007 |
| Slope after | -.049  [-.334, .235]  *t=*-.34, *p=*.734 | -.038  [-.325, .250]  *t=*-.26, *p=*.798 | -.011**  [-.019, -.004]  *t=*-2.88, *p=*.004 | -.011**  [-.019, -.004]  *t=*-2.91, *p=*.004 |
| Slope change | -.358  [-.749, .033]  *t=*-1.80, *p=*.072 | -.369  [-.762, .023]  *t=*-1.84, *p=*.065 | -.014**  [-.024, -.003]  *t=*-2.60, *p=*.009 | -.014*  [-.024, -.003]  *t=*-2.58, *p=*.010 |
| Individual vs. collective |  |  |  |  |
| Slope before | .072  [-.219, .363]  *t=*.48, *p=*.628 | .124  [-.179, .427]  *t=*.80, *p=*.423 | -.005  [-.012, .003]  *t=*-1.17, *p=*.243 | -.005  [-.013, .003]  *t=*-1.26, *p=*.208 |
| Immediate change | 1.279*  [.101, 2.457]  *t=*2.13, *p=*.033 | 1.363*  [.185, 2.541]  *t=*2.27, *p=*.023 | .070***  [.039, .102]  *t=*4.44, *p*<.001 | .067***  [.036, .098]  *t=*4.23, *p=*.000 |
| Slope after | -.136  [-.440, .167]  *t=*-.88, *p=*.379 | .015  [-.295, .324]  *t=*.09, *p=*.926 | -.008*  [-.016, .000]  *t=*-2.01, *p=*.044 | -.012**  [-.020, -.004]  *t=*-2.82, *p=*.005 |
| Slope change | -.208  [-.629, .213]  *t=*-.97, *p=*.332 | -.109  [-.532, .314]  *t=*-.51, *p=*.613 | -.004  [-.015, .007]  *t=*-.64, *p=*.520 | -.007  [-.018, .005]  *t=*-1.16, *p=*.245 |
| *R^2^* | .173 | .173 | .243 | .243 |
| Adjusted *R^2^* | .159 | .159 | .230 | .231 |
| Observations | 424,969 | 424,969 | 424,969 | 424,969 |
| *df* | 418,115 | 418,109 | 418,115 | 418,109 |

*Notes: ***p<.001 **p<.01 *p<.05. We multiply political diversity by 100 to interpret the estimates as absolute percentage point changes. We normalize content diversity to z-scores (the number of standard deviations from the mean). All regressions control for user fixed effects and the number of tweets per day. The statistical significance of regression coefficients is tested using two-sided t-tests. Confidence intervals (95%) are provided in brackets, along with the corresponding t-statistics, degrees of freedom, and exact p-values. More details can be found in Methods: Interrupted Time Series (ITS) Analysis.*

**Supplementary Table 27. Comparative Interrupted Time Series (CITS) Analyses**

| **Type of tagging** | **Individual Misinformation Tagging**  **(N=731,213)** | | **Collective Misinformation Tagging (N=97,539)** | |
| --- | --- | --- | --- | --- |
| **Outcome** | **Political Diversity (%)** | **Content Diversity (z)** | **Political Diversity (%)** | **Content Diversity (z)** |
| Control vs. Treatment |  |  |  |  |
| Slope before (*β_4_*) | .228***  [.118, .338]  *t=*4.05, *p*<.001 | .012***  [.010, .015]  *t=*8.25, *p*<.001 | -.150  [-.498, .197]  *t=*-.85, *p=*.397 | .019***  [.010, .028]  *t=*4.18, *p*<.001 |
| Immediate change (*β_5_*) | -.905***  [-1.344, -.465]  *t=*-4.03, *p*<.001 | -.018**  [-.030, -.006]  *t=*-3.01, *p=*.003 | .481  [-.953, 1.914]  *t=*.66, *p=*.511 | .100***  [.064, .136]  *t=*5.39, *p*<.001 |
| Slope change (*β_6_*) | .061  [-.094, .215]  *t=*.77, *p=*.441 | -.003  [-.007, .002]  *t=*-1.18, *p=*.238 | -.302  [-.810, .206]  *t=*-1.16, *p=*.244 | .015*  [.003, .028]  *t=*2.35, *p=*.019 |
| *R^2^* | .173 | .225 | .158 | .322 |
| Adjusted *R^2^* | .160 | .212 | .145 | .312 |
| Observations | 731,213 | 731,213 | 97,539 | 97,539 |
| *df* | 719,877 | 719,877 | 96,120 | 96,120 |

*Notes: ***p<.001 **p<.01 *p<.05. We multiply political diversity by 100 to interpret the estimates as absolute percentage point changes. We normalize content diversity to z-scores (the number of standard deviations from the mean). All regressions control for user fixed effects and the number of tweets per day. The statistical significance of regression coefficients is tested using two-sided t-tests. Confidence intervals (95%) are provided in brackets, along with the corresponding t-statistics, degrees of freedom, and exact p-values.*

**Supplementary Table 28. Effects of Individual and Collective Tags after Including Autoregressive Terms**

| **Outcome** | **Political diversity (%)** | | **Content diversity (z)** | |
| --- | --- | --- | --- | --- |
| **Adjustment** | **Initial results**  **(*N*=424,969)** | **Including an Autoregressive Term (*N* = 418,122)** | **Initial results**  **(*N*=424,969)** | **Including an Autoregressive Term (*N* = 418,122)** |
| Individual |  |  |  |  |
| Slope before | .237***  [.125, .349]  *t=*4.14, *p*<.001 | .224***  [.109, .338]  *t=*3.84, *p*<.001 | .007***  [.004, .010]  *t=*4.79, *p*<.001 | .007***  [.004, .010]  *t=*4.45, *p*<.001 |
| Immediate change | -1.009***  [-1.447, -.571]  *t=*-4.52, *p*<.001 | -.900***  [-1.336, -.464]  *t=*-4.050, *p*<.001 | -.030***  [-.042, -.019]  *t=*-5.10, *p*<.001 | -.025***  [-.036, -.013]  *t=*-4.24, *p*<.001 |
| Slope after | .087  [-.020, .194]  *t=*1.60, *p=*.110 | .082  [-.024, .188]  *t=*1.52, *p=*.128 | -.003  [-.006, .000]  *t=*-1.95, *p=*.051 | -.002  [-.005, .001]  *t=*-1.49, *p=*.137 |
| Slope change | -.150  [-.306, .006]  *t=*-1.89, *p=*.059 | -.141  [-.298, .015]  *t=*-1.77, *p=*.077 | -.010***  [-.014, -.006]  *t=*-4.79, *p*<.001 | -.009***  [-.013, -.005]  *t=*-4.25, *p<.*001 |
| Collective |  |  |  |  |
| Slope before | .309*  [.041, .578]  *t=*2.26, *p=*.024 | .230  [-.042, .501]  *t=*1.66, *p=*.097 | .003  [-.004, .010]  *t=*.74, *p=*.461 | .004  [-.003, .011]  *t=*1.13, *p=*.257 |
| Immediate change | .270  [-.824, 1.363]  *t=*.48, *p=*.629 | .271  [-.815, 1.357]  *t=*.49, *p=*.625 | .040**  [.012, .069]  *t=*2.74, *p=*.006 | .029*  [.000, .057]  *t=*1.98, *p=*.047 |
| Slope after | -.049  [-.334, .235]  *t=*-.34, *p=*.734 | -.023  [-.304, .258]  *t=*-.16, *p=*.873 | -.011**  [-.019, -.004]  *t=*-2.88, *p=*.004 | -.009*  [-.017, -.002]  *t=*-2.43, *p=*.015 |
| Slope change | -.358  [-.749, .033]  *t=*-1.80, *p=*.072 | -.253  [-.644, .138]  *t=*-1.27, *p=*.205 | -.014**  [-.024, -.003]  *t=*-2.60, *p=*.009 | -.013*  [-.023, -.003]  *t=*-2.54, *p=*.011 |
| Individual vs. collective |  |  |  |  |
| Slope before | .072  [-.219, .363]  *t=*.48, *p=*.628 | .006  [-.288, .301]  *t=*.04, *p=*.967 | -.005  [-.012, .003]  *t=*-1.17, *p=*.243 | -.003  [-.010, .005]  *t=*-.68, *p=*.496 |
| Immediate change | 1.279*  [.101, 2.457]  *t=*2.13, *p=*.033 | 1.171*  [.001, 2.341]  *t=*1.96, *p=*.050 | .070***  [.039, .102]  *t=*4.44, *p*<.001 | .053**  [.023, .084]  *t=*3.42, *p=*.001 |
| Slope after | -.136  [-.440, .167]  *t=*-.88, *p=*.379 | -.105  [-.406, .195]  *t=*-.69, *p=*.492 | -.008*  [-.016, .000]  *t=*-2.01, *p=*.044 | -.007  [-.015, .001]  *t=*-1.75, *p=*.080 |
| Slope change | -.208  [-.629, .213]  *t=*-.97, *p=*.332 | -.112  [-.533, .309]  *t=*-.52, *p=*.604 | -.004  [-.015, .007]  *t=*-.64, *p=*.520 | -.004  [-.015, .007]  *t=*-.78, *p=*.438 |
| *R^2^* | .173 | .188 | .243 | .269 |
| Adjusted *R^2^* | .159 | .176 | .230 | .258 |
| Observations | 424,969 | 418,122 | 424,969 | 418,122 |
| *df* | 418,115 | 411,919 | 418,115 | 411,919 |

*Notes: ***p<.001 **p<.01 *p<.05. We multiply political diversity by 100 to interpret the estimates as absolute percentage point changes. We normalize content diversity to z-scores (the number of standard deviations from the mean). All regressions control for user fixed effects and the number of tweets per day. The statistical significance of regression coefficients is tested using two-sided t-tests. Confidence intervals (95%) are provided in brackets, along with the corresponding t-statistics, degrees of freedom, and exact p-values. More details can be found in Methods: Interrupted Time Series (ITS) Analysis.*

**Supplementary Table 29. Topic Distribution of Tweets Corrected by Individual and Collective Tags Before and After Propensity Score Weighting (PSW)**

|  |  | **Before PSW** | | **After PSW** | |
| --- | --- | --- | --- | --- | --- |
| **Topic ID** | **Top keywords** | **Individual (%)** | **Collective (%)** | **Individual (%)** | **Collective (%)** |
| 1 | covid, vaccine, vaccines, vaccinated | 33.81% | 17.25% | 32.41% | 32.41% |
| 2 | fact, just, lies, true | 24.70% | 15.72% | 23.94% | 23.94% |
| 3 | gun, state, state lines, lines | 10.02% | 12.66% | 10.24% | 10.24% |
| 4 | projection, lie republican, monster, fyi | 9.13% | 7.64% | 9.00% | 9.00% |
| 5 | tax, biden, inflation, bernie | 9.41% | 5.46% | 9.08% | 9.08% |
| 6 | ukraine, russia, biden, putin | 3.37% | 11.14% | 4.03% | 4.03% |
| 7 | votes, election, voters, vote | 1.64% | 25.11% | 3.62% | 3.62% |
| 8 | hillary, thomas, clinton, just | 4.28% | 0.87% | 3.99% | 3.99% |
| 9 | turkey, earthquake, climate, al gore | 3.64% | 4.15% | 3.68% | 3.68% |

*Notes: Top keywords have been identified by counting the most frequent words within tweets corresponding to each topic, following the exclusion of stopwords. Individual (%) column indicates the proportion of a particular topic out of the misinformation corrected through individual tagging. Collective (%) column indicates the proportion of a particular topic out of the misinformation corrected through collective tagging. Proportion values were calculated based on the top nine topics.*

**Supplementary Fig. 1. Examples of Individual and Collective Misinformation Tagging. a,** An instance of an individual misinformation tagging regarding COVID-19 vaccination through a reply. **b,** An instance of a collective misinformation tagging regarding COVID-19 vaccination, which is shown above other users’ replies. Usernames and profiles have been anonymized. Following Twitter's privacy policy, we provide the manually rephrased tweets to ensure that user identities remain confidential.


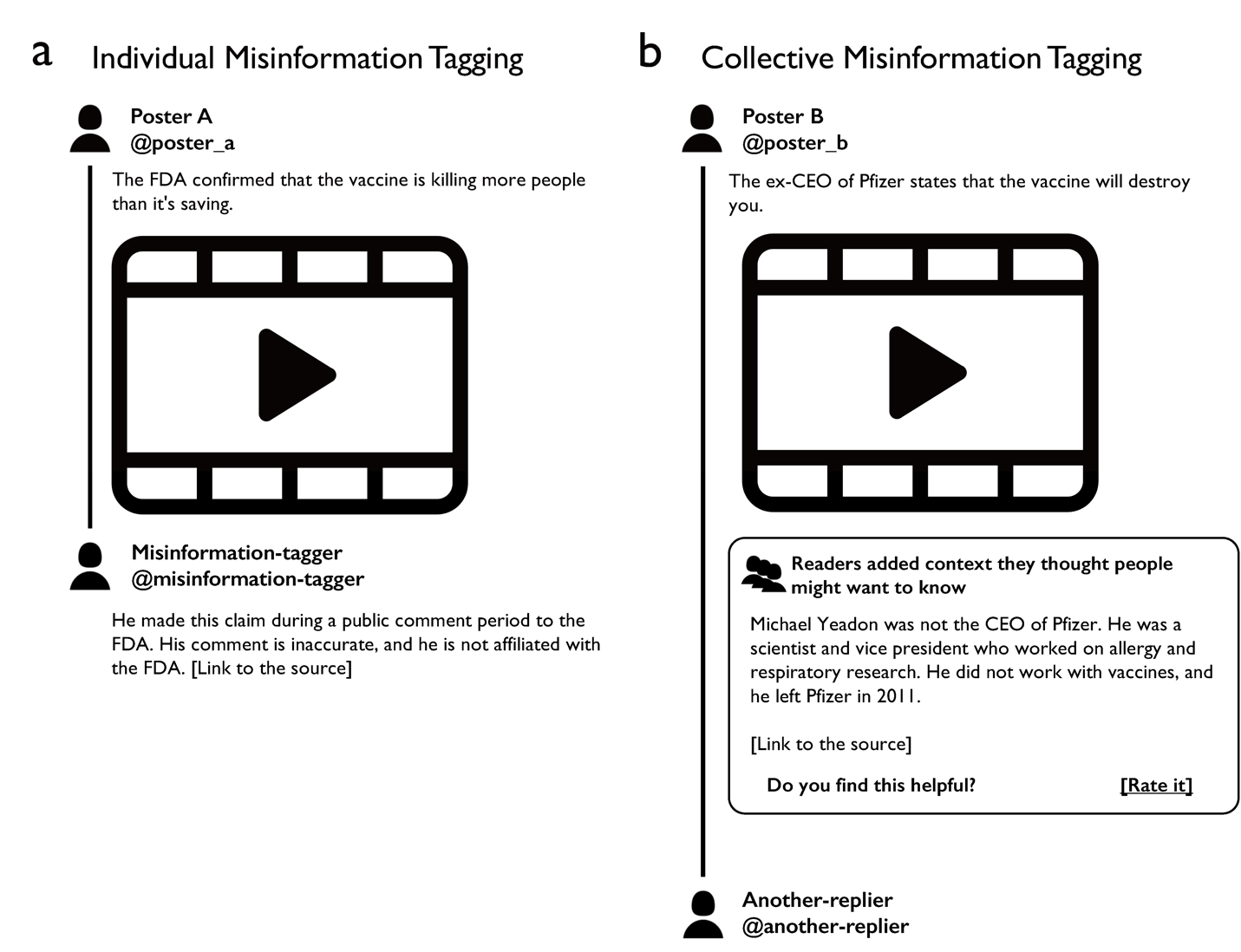


**Supplementary Fig. 2. An example of a pair of matched tweets and tags in delayed feedback (DF) analysis.** Here, a pair of corrected tweets shows similar characteristics: they are about the same topic (i.e., the earthquake in Türkiye) and were posted at approximately the same time (tweet in the control group: February 6, 2023, 9:53 AM; tweet in the treatment group: February 6, 2023, 7:21 AM). Nevertheless, they were corrected at very different times (tag in the control group: February 9, 2023, 5:10 AM; tag in the treatment group: February 6, 2023, 6:09 PM), constituting a gap of 53 hours. Following Twitter's privacy policy, we provide the manually rephrased tweets and posting times to ensure that user identities remain confidential.


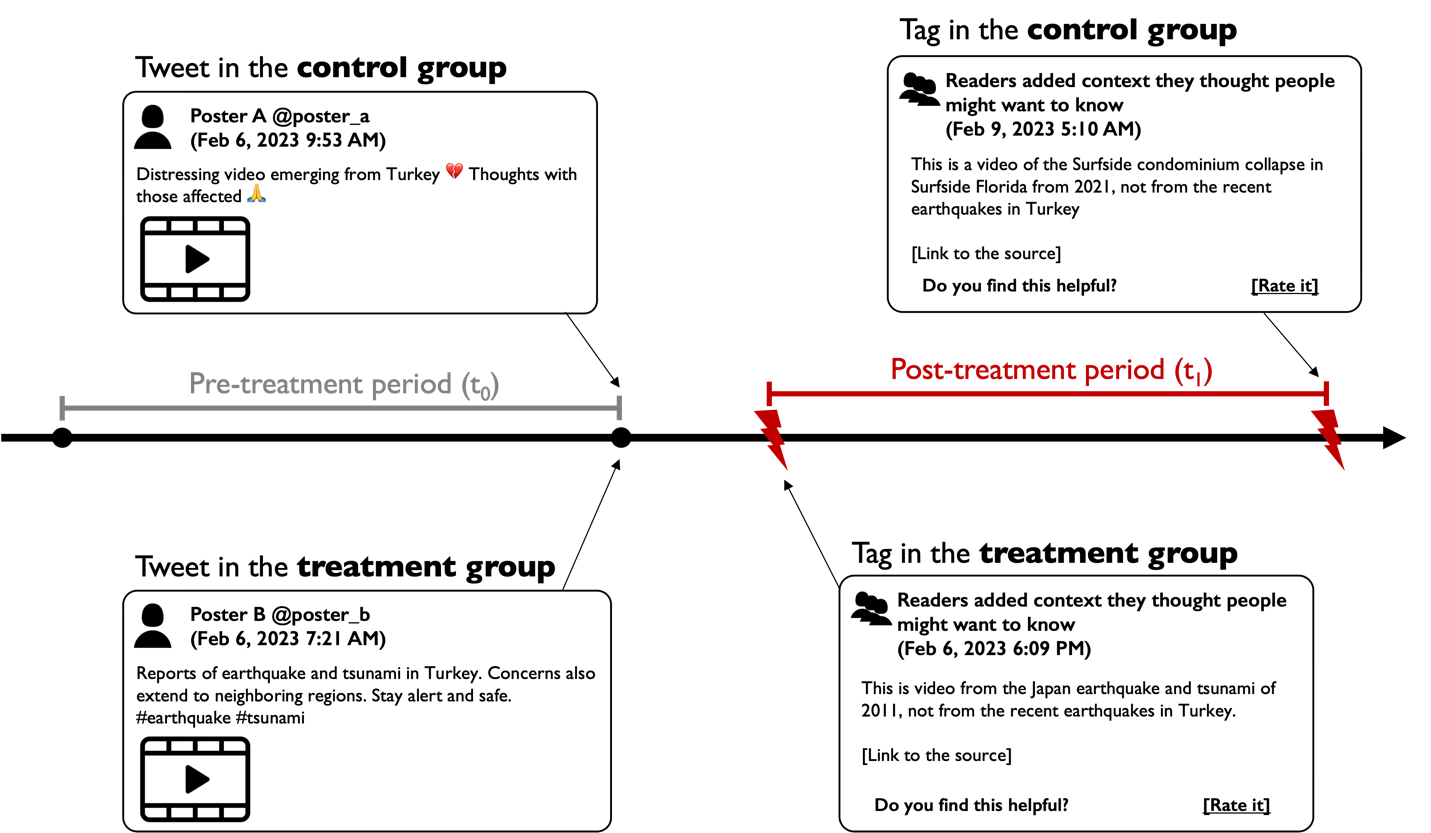


**Supplementary Fig. 3. Distribution of the Log-Transformed Number of Followers of Tagged Users.** The blue line indicates the distribution of the log-transformed number of followers among posters fact-checked by individual tags. The orange line indicates the distribution among posters fact-checked by collective tags. The gray dashed line indicates the average number of followers among posters fact-checked by individual tags (i.e., 2,967).


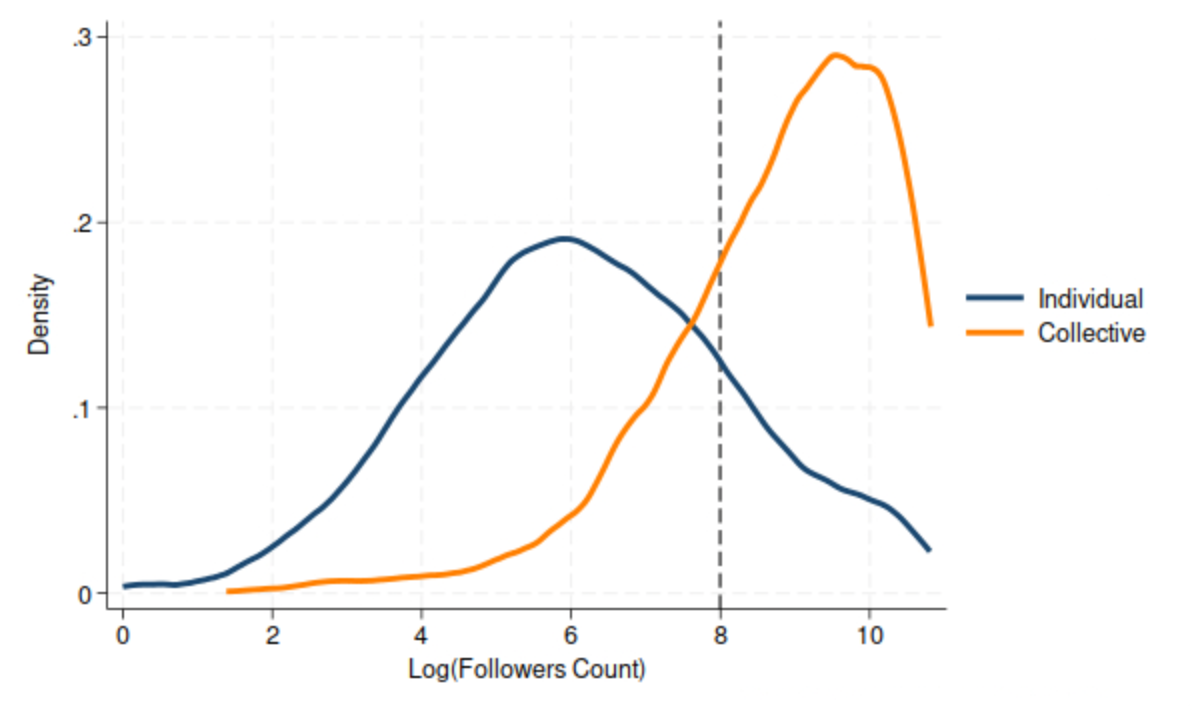


**Supplementary Fig. 4. Distribution of Tagged Posters’ Political Stances.** The political stance scores range from -1, indicating a left-leaning stance, to 1, indicating a right-leaning stance. The average political stance is .166 (SD = .372), indicating a tendency for a larger proportion of users to lean to the right.


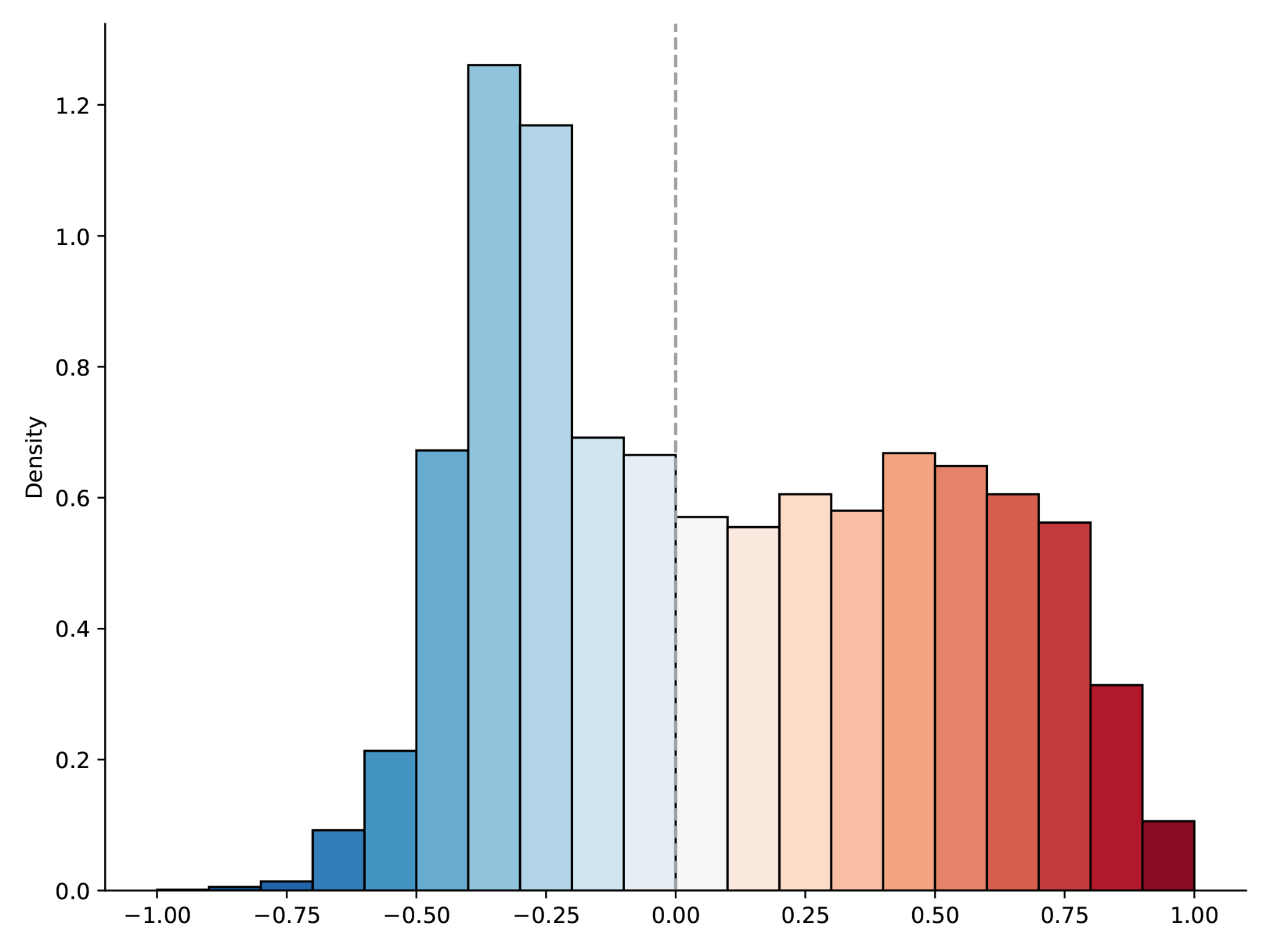


**Supplementary Fig. 5. Tweet Embedding Space.** This figure presents a two-dimensional UMAP (Uniform Manifold Approximation and Projection) visualization, illustrating the embeddings of a randomly selected subset of 10,000 tweets posted before and after misinformation tagging. We show the largest cluster identified by HDBSCAN (Hierarchical Density-Based Spatial Clustering of Applications with Noise).


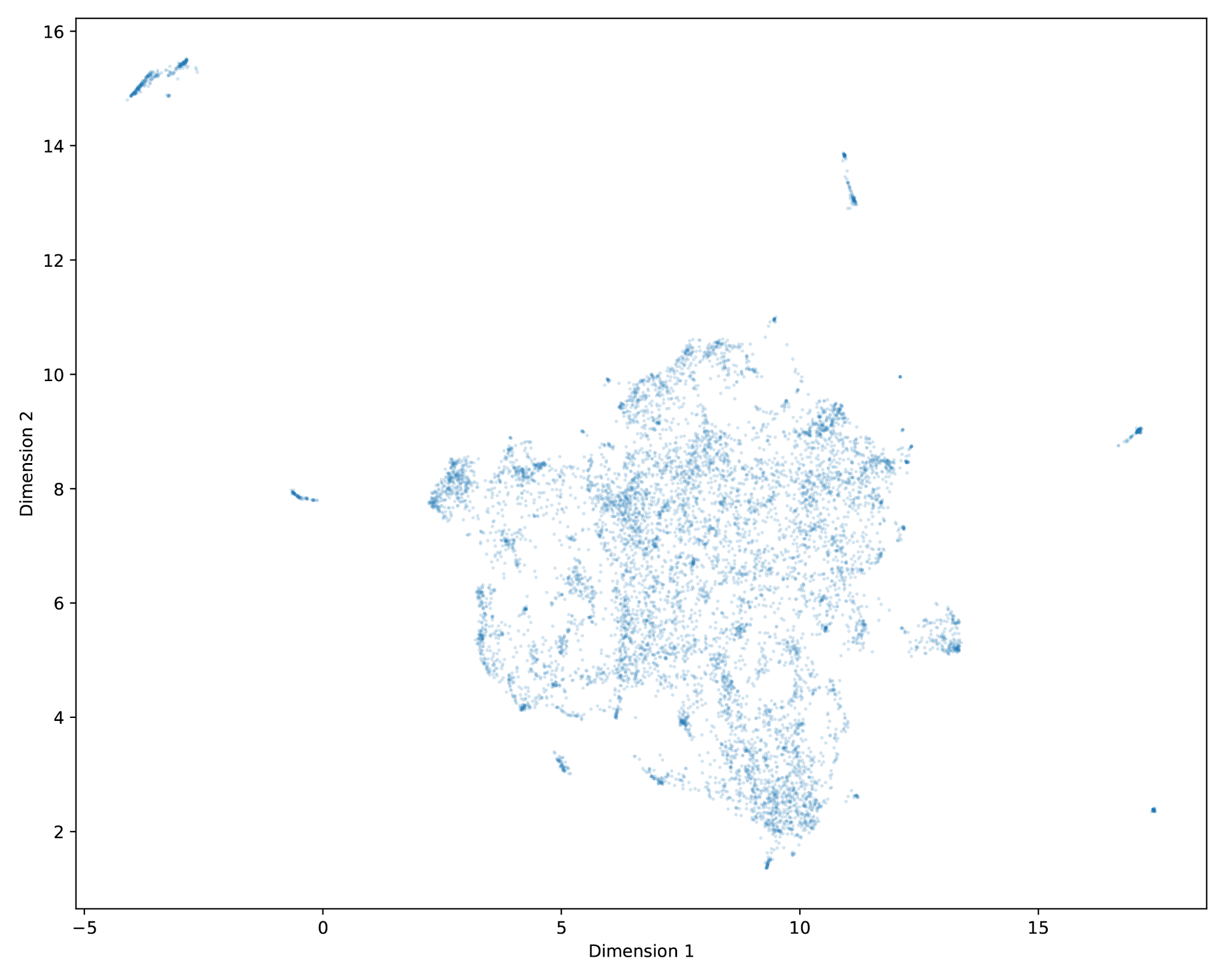


**Supplementary Fig. 6. Trends of Political and Content Diversity Before Receiving Any Misinformation Tag in the Delayed Feedback (DF) Analysis.** The *x*-axis represents the timeline of tweets, indicating the number of days before receiving any misinformation tag. The *y*-axis shows political and content diversity, with dots representing the average diversity score for each day, controlling for time-invariant between-user characteristics (i.e., controlling for user-level fixed effects). Linear fit lines illustrate trends for both treatment and control groups, allowing us to assess the parallel trends assumption.

| 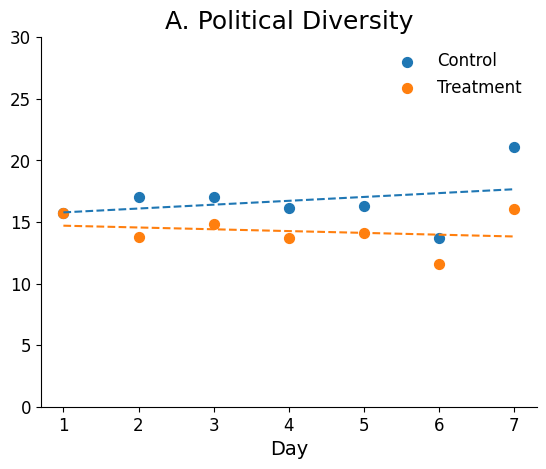 | 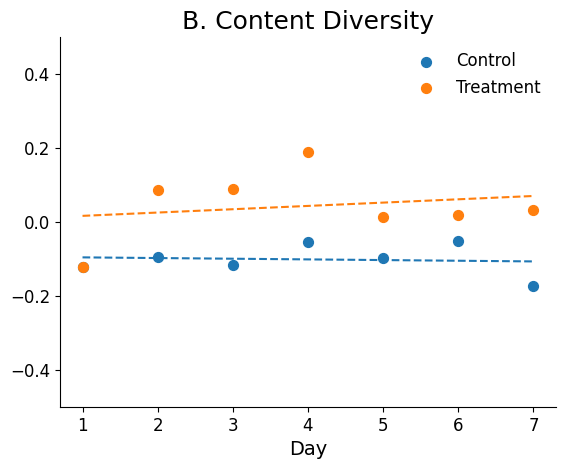 |
| --- | --- |

**Supplementary Fig. 7. Average Baseline Changes of Political and Content Diversity Obtained from the Control Group in the Delayed Feedback (DF) Analysis.** The x-axis denotes *t_0_* (pre-treatment period) and *t_1_* (post-treatment period). The y-axis denotes political and content diversity, with dots capturing the average diversity score of the corresponding period, and error bars indicating 95% confidence intervals.

**
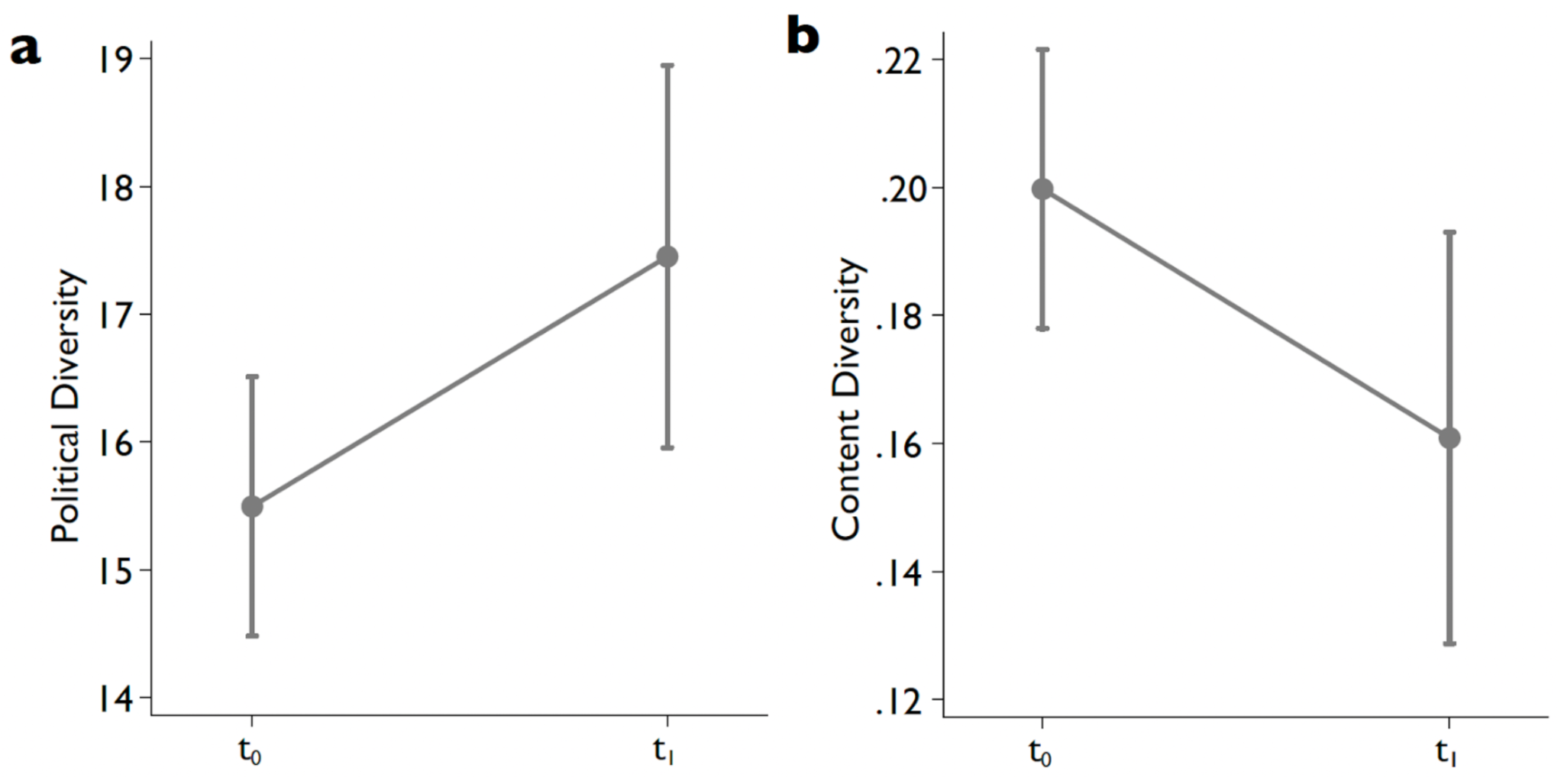
**

**Supplementary Fig. 8. Political and Content Proximity to Taggers Changes with the Intervention of Individual Misinformation Tagging.** Results from Interrupted Time Series (ITS) analysis. The *x*-axis denotes the timeline of tweets posted before and after tagging, with negative values indicating the number of weeks before posting tagged tweets and positive values indicating the number of weeks after. The *y*-axis denotes political and content proximity to taggers, with dots capturing the average diversity score of the corresponding week, and error bars indicating 95% confidence intervals.

**
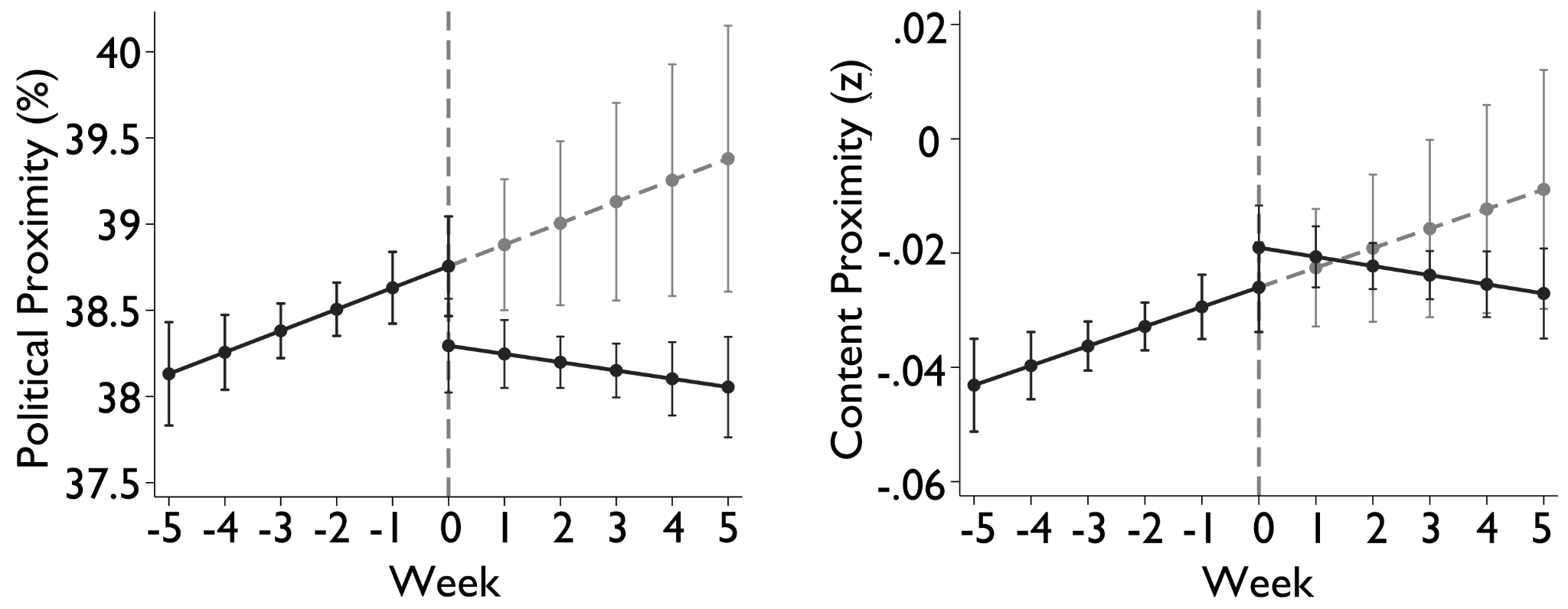
**

**Supplementary Fig. 9. Semantic differences between tweets tagged with individual and collective tags before and after propensity score weighting (PSW). a,** A two-dimensional PCA (principal component analysis) visualization illustrating the semantic embeddings of tweets tagged with individual or collective tags. **b,** The average standardized difference between “tweets tagged by individual tags” and “tweets tagged by collective tags” before and after PSW. The standardized difference is calculated for each dimension of the semantic embeddings, and the average is taken. Error bars represent the 95% confidence interval.

| 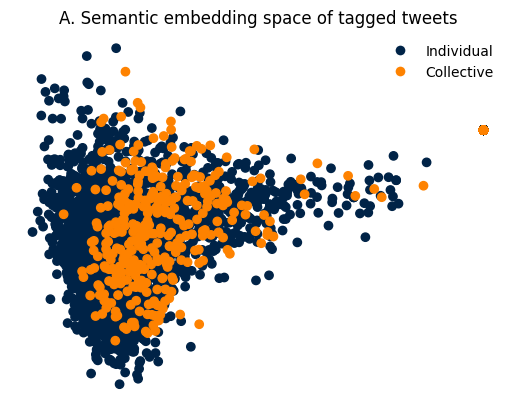 | 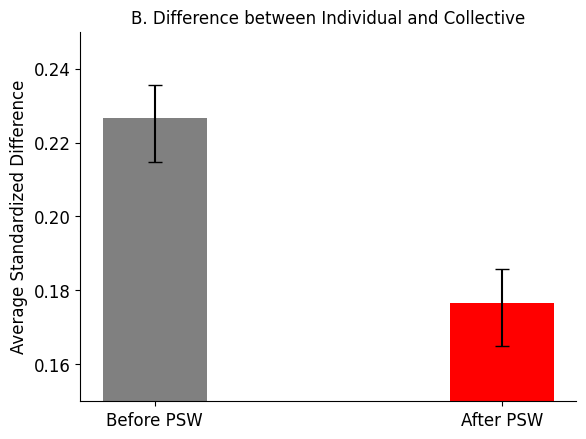 |
| --- | --- |

## **References**

1. Li, F., Morgan, K. L. & Zaslavsky, A. M. Balancing Covariates via Propensity Score Weighting. *J. Am. Stat. Assoc.* **113**, 390–400 (2018).

2. Allen, J., Martel, C. & Rand, D. G. Birds of a feather don’t fact-check each other: Partisanship and the evaluation of news in Twitter’s Birdwatch crowdsourced fact-checking program. in *Proceedings of the 2022 CHI Conference on Human Factors in Computing Systems* 1–19 (Association for Computing Machinery, 2022).

3. Barberá, P. Birds of the Same Feather Tweet Together: Bayesian Ideal Point Estimation Using Twitter Data. *Polit. Anal.* **23**, 76–91 (2015).

4. Wojcik, S. *et al.* Birdwatch: Crowd Wisdom and Bridging Algorithms can Inform Understanding and Reduce the Spread of Misinformation. *Preprint at https://arxiv.org/abs/2210.15723 (2022).*

5. Flamino, J. *et al.* Political polarization of news media and influencers on Twitter in the 2016 and 2020 US presidential elections. *Nat Hum Behav* **7**, 904–916 (2023).
