# Supplementary material for: Differential impact from individual versus collective misinformation tagging on the diversity of Twitter (X) information engagement and mobility
Source: Nat Commun. 2025 Jan 24;16:973. doi: 10.1038/s41467-025-55868-0 (PMC11760358; doi:10.1038/s41467-025-55868-0)
Supplement: Supplementary file 2 — Reporting Summary [file 41467_2025_55868_MOESM2_ESM.pdf]

# nature portfolio

Corresponding author(s): James Evans

Last updated by author(s): Oct 7, 2024

# Reporting Summary

Nature Portfolio wishes to improve the reproducibility of the work that we publish. This form provides structure for consistency and transparency in reporting. For further information on Nature Portfolio policies, see our Editorial Policies and the Editorial Policy Checklist.

## Statistics

For all statistical analyses, confirm that the following items are present in the figure legend, table legend, main text, or Methods section.

| n/a | Confirmed | |
|---|---|---|
| ☐ | ☒ | The exact sample size ($n$) for each experimental group/condition, given as a discrete number and unit of measurement |
| ☐ | ☒ | A statement on whether measurements were taken from distinct samples or whether the same sample was measured repeatedly |
| ☐ | ☒ | The statistical test(s) used AND whether they are one- or two-sided<br>*Only common tests should be described solely by name; describe more complex techniques in the Methods section.* |
| ☐ | ☒ | A description of all covariates tested |
| ☐ | ☒ | A description of any assumptions or corrections, such as tests of normality and adjustment for multiple comparisons |
| ☐ | ☒ | A full description of the statistical parameters including central tendency (e.g. means) or other basic estimates (e.g. regression coefficient) AND variation (e.g. standard deviation) or associated estimates of uncertainty (e.g. confidence intervals) |
| ☐ | ☒ | For null hypothesis testing, the test statistic (e.g. $F$, $t$, $r$) with confidence intervals, effect sizes, degrees of freedom and $P$ value noted<br>*Give P values as exact values whenever suitable.* |
| ☐ | ☒ | For Bayesian analysis, information on the choice of priors and Markov chain Monte Carlo settings |
| ☐ | ☒ | For hierarchical and complex designs, identification of the appropriate level for tests and full reporting of outcomes |
| ☐ | ☒ | Estimates of effect sizes (e.g. Cohen's $d$, Pearson's $r$), indicating how they were calculated |

*Our web collection on statistics for biologists contains articles on many of the points above.*

## Software and code

Policy information about availability of computer code

| | |
|---|---|
| Data collection | Using Twitter API v2.0 with academic research access, we collected tweets (i.e., Twitter posts) posted by Twitter users who had been targeted by individual or collective misinformation tagging. |
| Data analysis | We used Stata 17 and Python 3.9 to analyze the data. The code necessary to replicate our results is available at https://doi.org/10.17605/OSF.IO/TXGSR. |

For manuscripts utilizing custom algorithms or software that are central to the research but not yet described in published literature, software must be made available to editors and reviewers. We strongly encourage code deposition in a community repository (e.g. GitHub). See the Nature Portfolio guidelines for submitting code & software for further information.

## Data

Policy information about availability of data

All manuscripts must include a data availability statement. This statement should provide the following information, where applicable:

- Accession codes, unique identifiers, or web links for publicly available datasets
- A description of any restrictions on data availability
- For clinical datasets or third party data, please ensure that the statement adheres to our policy

We comply with the terms of service of our data source, Twitter (X). We used data from Twitter API v2.0 with officially granted academic research access, which permits us to use their data for non-commercial, research purposes (https://developer.twitter.com/en/developer-terms/commercial-terms). We conduct aggregate analysis of anonymized Twitter content that does not store any personal data (for example, user IDs, usernames, and other identifiers), which is explicitly permitted

in their terms of service (See sensitive information; https://developer.twitter.com/en/developer-terms/more-on-restricted-use-cases; we never link inferred political attitudes and actual user IDs). We also match Twitter accounts with popular organizations (e.g., CNN) or identify the probability that a particular account is a bot, using the public data that Twitter explicitly lists and permits in their terms of service (See Off-Twitter matching; https://developer.twitter.com/en/developer-terms/more-on-restricted-use-cases).

In compliance with Twitter's privacy policy, we are unable to disclose individual-level Twitter user information or the contents of tweets. Therefore, we offer anonymized data for reproducing our findings. We aggregate the analysis of anonymized tweets in our paper without depending on any user-specific information (for example, user IDs, usernames, and other identifiers). This data is available at: https://doi.org/10.17605/OSF.IO/TXGSR

## Research involving human participants, their data, or biological material

Policy information about studies with human participants or human data. See also policy information about sex, gender (identity/presentation), and sexual orientation and race, ethnicity and racism.

| | |
|---|---|
| Reporting on sex and gender | The information regarding the sex and gender of Twitter users is not disclosed, and these factors are not considered in our study design. The findings are not specific to any particular sex or gender. |
| Reporting on race, ethnicity, or other socially relevant groupings | The only social grouping considered in our study is political stance, as we analyze whether misinformation tagging impacts the political echo chamber. This has been measured based on the proportion of left- and right-leaning news sources referenced in a user's tweets (refer to Methods - Political Diversity). Note that we conduct an aggregate analysis of political stances using anonymized Twitter content, which is explicitly permitted in Twitter terms of service (See sensitive information; https://developer.twitter.com/en/developer-terms/more-on-restricted-use-cases). We account for individual-level fixed effects to adjust for any time-invariant characteristics of users. |
| Population characteristics | A larger proportion of users lean to the right. In our data, political stance scores range from -1, indicating a left-leaning stance, to +1, indicating a right-leaning stance. The average political stance score is 0.166 (SD = 0.372), suggesting a tendency towards a right-leaning bias among fact-checked users. |
| Recruitment | In our study, participants were not actively recruited; instead, we passively collected publicly available tweets posted by users who had been targeted by individual or collective misinformation tagging. |
| Ethics oversight | The use of the publicly available Twitter data does not constitute research with human subjects because there is no direct interaction with any individual, and no identifiable private information is used. Because all data for the study analyzed was publicly available, it was not deemed to meet the definition of identifiable private information per 45 CFR 46.102(e)4-5 by the University of Chicago Social and Behavioral Sciences IRB.<br><br>This research study received a determination from the University of Chicago Social & Behavioral Sciences Institutional Review Board that the study is not considered human subjects research and does not require review (Institutional Review Board Protocol IRB24-0051). |

Note that full information on the approval of the study protocol must also be provided in the manuscript.

# Field-specific reporting

Please select the one below that is the best fit for your research. If you are not sure, read the appropriate sections before making your selection.

☐ Life sciences ☒ Behavioural & social sciences ☐ Ecological, evolutionary & environmental sciences

For a reference copy of the document with all sections, see nature.com/documents/nr-reporting-summary-flat.pdf

# Behavioural & social sciences study design

All studies must disclose on these points even when the disclosure is negative.

| | |
|---|---|
| Study description | We conducted quantitative research using observational, non-experimental, and publicly available data from Twitter. |
| Research sample | We collected tweets (i.e., Twitter posts) posted by Twitter users who had been targeted by individual or collective misinformation tagging. A larger proportion of users tend to reference right-leaning news articles than left-leaning news articles, suggesting a right-leaning bias among fact-checked users. |
| Sampling strategy | We collected data from all Twitter users targeted by misinformation tagging, and no sampling strategy (e.g., random, snowball, stratified, convenience) was involved. |
| Data collection | We used Twitter API v2.0 with academic research access to collect tweets (i.e., Twitter posts) posted by users. |
| Timing | We focused on Twitter users targeted by individual misinformation tagging from October 1, 2021, to March 25, 2022, and collective tagging from December 19, 2022, to March 31, 2023 (i.e., after Community Notes were released to the public globally). Then, we collected these users' historical tweets which span two months before posting tagged tweets and two months after the exposure of misinformation tagging. |
| Data exclusions | Using Twitter API v2.0 with academic research access, we collected Twitter data to explore the effects of individual and collective |

| | |
|---|---|
| | misinformation tagging. First, we identified 9,372 users targeted by individual misinformation tagging from 2021/10/1 to 2022/3/25. We selected users whose tweets received fact-checking replies that contain URLs to fact-checking articles from "politifact.com." Second, we identified 1,465 users targeted by collective tagging from 2022/12/19 to 2023/3/31, when Community Notes were made public to Twitter users globally.<br><br>Due to the rate limit of Twitter API, we only collected data from regular Twitter users, excluding organizations' and celebrities' accounts with 50,000 or more followers. Additionally, to focus on individual users, rather than organizational accounts (e.g., CNN, Fox News, etc), we removed 1,659 users identified as organization accounts by the M3Inference library. We further removed 1,445 users who were fact-checked more than once within the period of data collection to avoid the potential for them to become desensitized for repeated fact-checks. After filtering the data, our final dataset included 7,733 users, where 6,760 users were targeted by individual misinformation tagging and 973 users were targeted by collective misinformation tagging. |
| Non-participation | We collected tweets that are already publicly available, so there are no instances of dropout or declined participation. |
| Randomization | Participants were not randomly allocated into experimental groups. |

# Reporting for specific materials, systems and methods

We require information from authors about some types of materials, experimental systems and methods used in many studies. Here, indicate whether each material, system or method listed is relevant to your study. If you are not sure if a list item applies to your research, read the appropriate section before selecting a response.

## Materials & experimental systems

| n/a | Involved in the study | |
|---|---|---|
| ☒ | ☐ | Antibodies |
| ☒ | ☐ | Eukaryotic cell lines |
| ☒ | ☐ | Palaeontology and archaeology |
| ☒ | ☐ | Animals and other organisms |
| ☒ | ☐ | Clinical data |
| ☒ | ☐ | Dual use research of concern |
| ☒ | ☐ | Plants |

## Methods

| n/a | Involved in the study | |
|---|---|---|
| ☒ | ☐ | ChIP-seq |
| ☒ | ☐ | Flow cytometry |
| ☒ | ☐ | MRI-based neuroimaging |

# Plants

| | |
|---|---|
| Seed stocks | *Report on the source of all seed stocks or other plant material used. If applicable, state the seed stock centre and catalogue number. If plant specimens were collected from the field, describe the collection location, date and sampling procedures.* |
| Novel plant genotypes | *Describe the methods by which all novel plant genotypes were produced. This includes those generated by transgenic approaches, gene editing, chemical/radiation-based mutagenesis and hybridization. For transgenic lines, describe the transformation method, the number of independent lines analyzed and the generation upon which experiments were performed. For gene-edited lines, describe the editor used, the endogenous sequence targeted for editing, the targeting guide RNA sequence (if applicable) and how the editor was applied.* |
| Authentication | *Describe any authentication procedures for each seed stock used or novel genotype generated. Describe any experiments used to assess the effect of a mutation and, where applicable, how potential secondary effects (e.g. second site T-DNA insertions, mosiacism, off-target gene editing) were examined.* |
